# Supplementary material for: Study on the Neuroprotective, Radical-Scavenging and MAO-B Inhibiting Properties of New Benzimidazole Arylhydrazones as Potential Multi-Target Drugs for the Treatment of Parkinson’s Disease
Source: Antioxidants (Basel). 2022 Apr 29;11(5):884. doi: 10.3390/antiox11050884 (PMC9138090; doi:10.3390/antiox11050884)
Supplement: Supplementary file 1 [file antioxidants-11-00884-s001.zip › antioxidants-1660263-supplementary.pdf]

Supplementary material

Study on the neuroprotective, radical-scavenging and MAO-B inhibiting properties of new benzimidazole arylhydrazones as potential multi-target drugs for the treatment of Parkinson's disease

Neda Anastassova, Denitsa Aluani, Nadya Hristova-Avakumova, Virginia Tzankova, Magdalena Kondeva-Burdina, Miroslav Rangelov, Nadezhda Todorova, Denitsa Yancheva

Contents

1. ^{1}H NMR spectra showing the ratio of the conformers
2. ^{1}H NMR and ^{13}C NMR spectra of the synthesized compounds
3. ATR-IR spectra of the synthesized compounds
4. Three-dimensional (3D) representation of lower-energy docking poses of **3h** in the MAO-B cavity

1. ^{1}H NMR spectra showing the ratio of the conformers

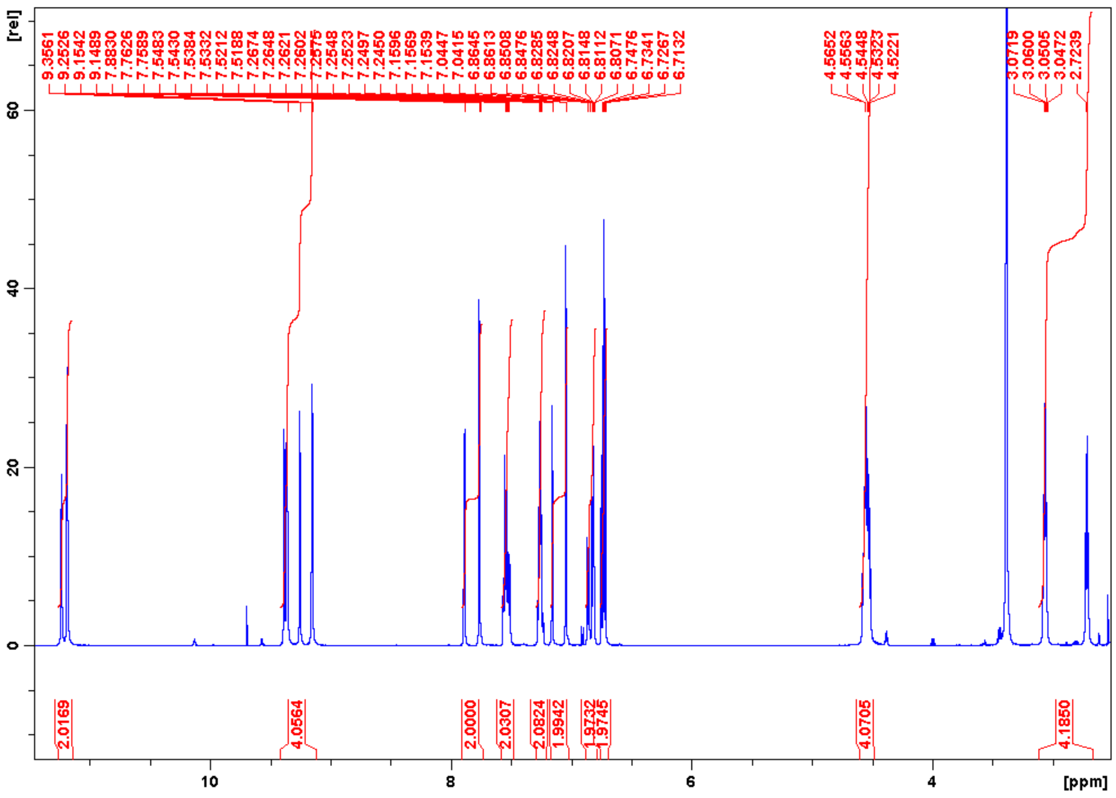

Figure S1. ^{1}H NMR spectrum of **3h**

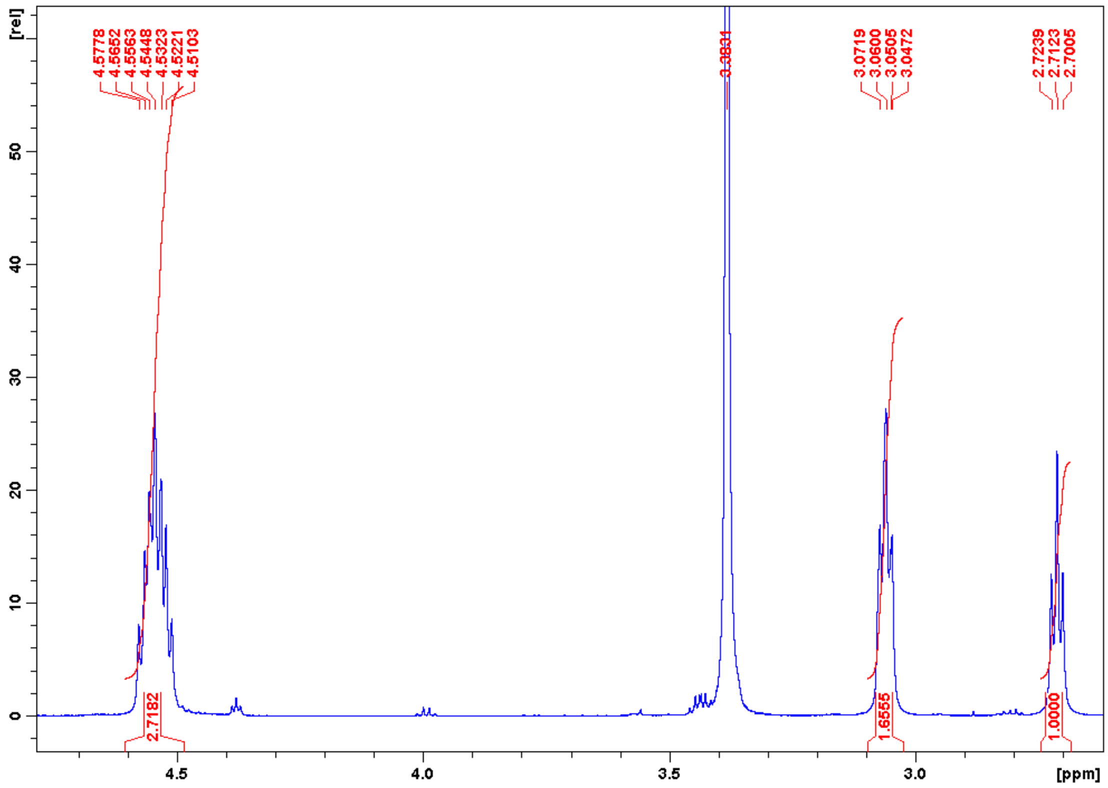

Figure S2. Ratio of the multiple sets of resonances in the ^{1}H-NMR spectrum of the -O-CH_2- group of **3h**

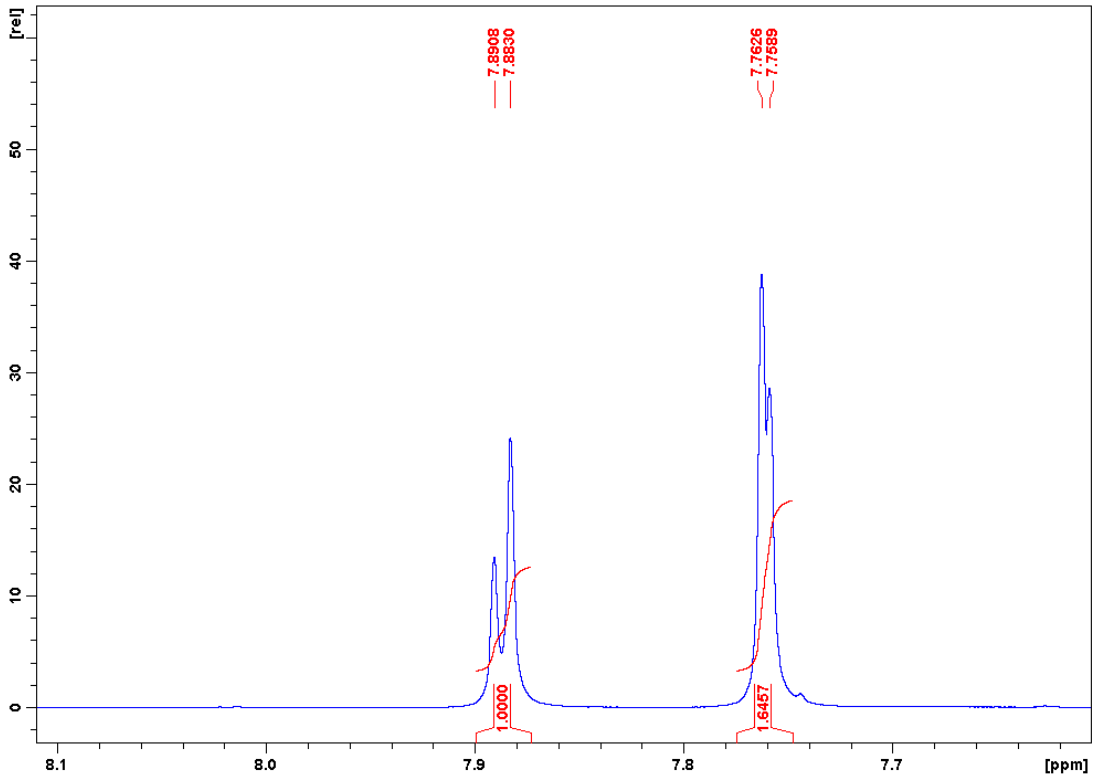

Figure S3. Ratio of the doublets for the –CH=N- group of the ^{1}H-NMR spectrum of **3h**

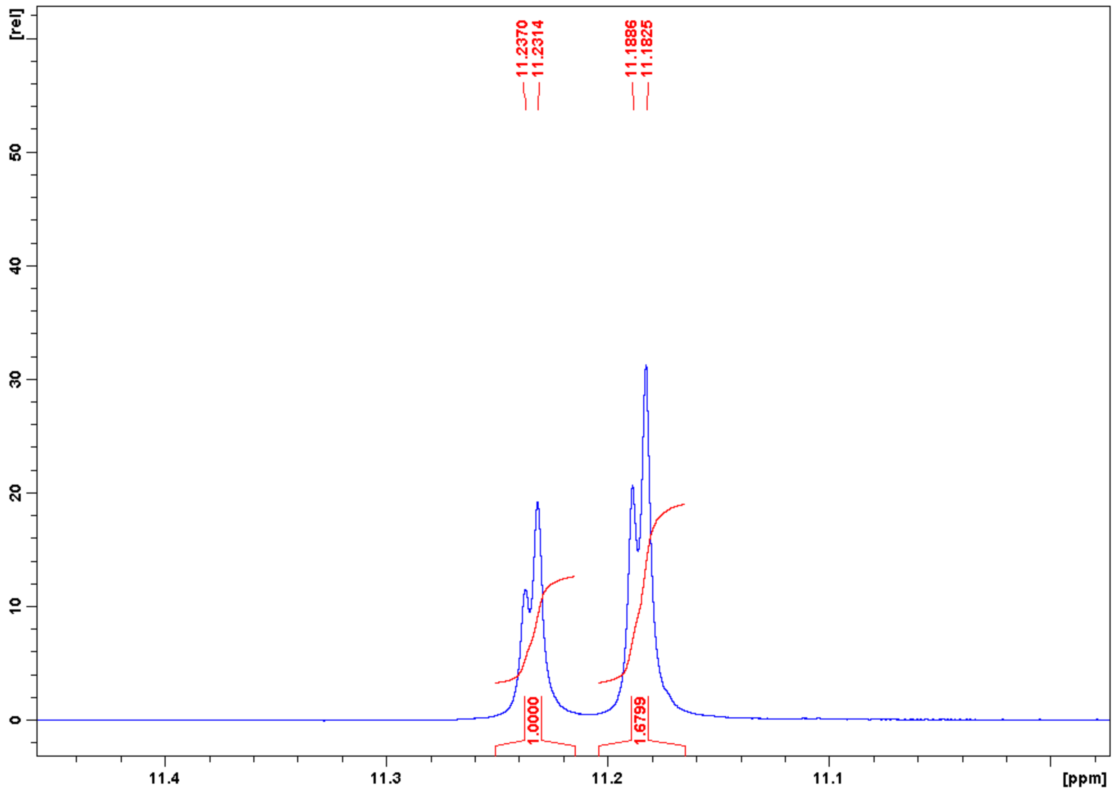

Figure S4. Ratio of the doublets for the –NH- group of the ^{1}H-NMR spectrum of **3h**

2. NMR spectra of the synthesized compounds

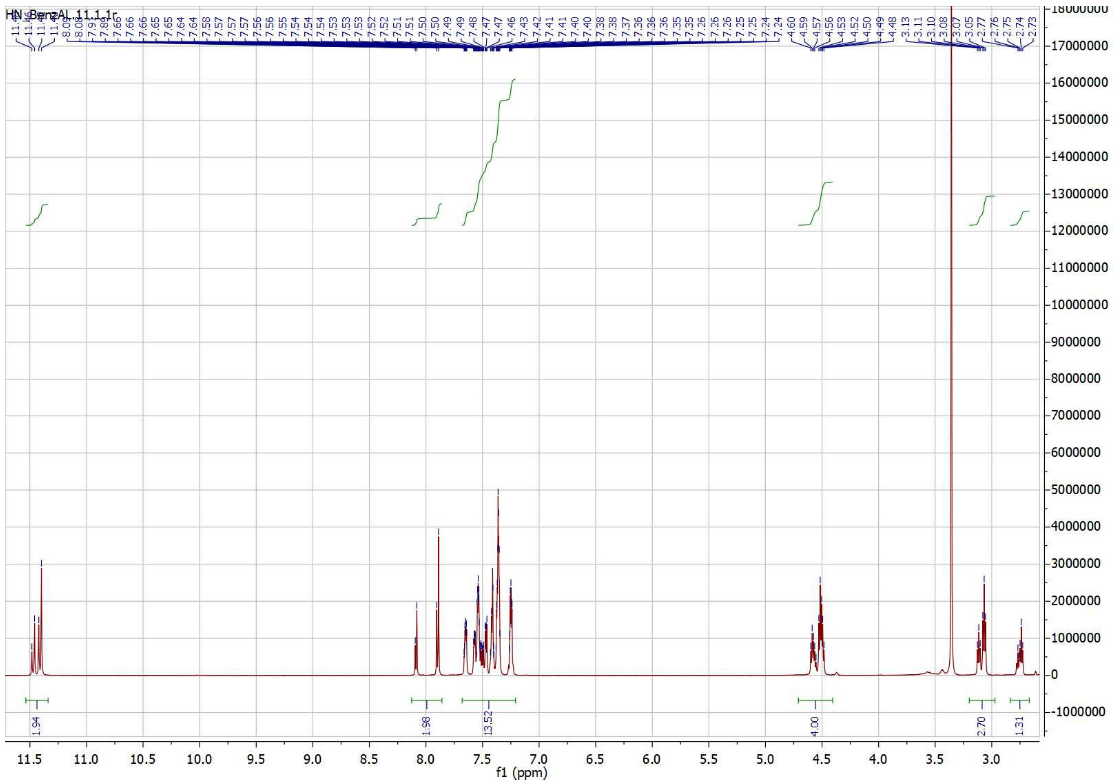

Figure S5. ^{1}H NMR spectrum of **3a** (600 MHz, DMSO-d$_6$).

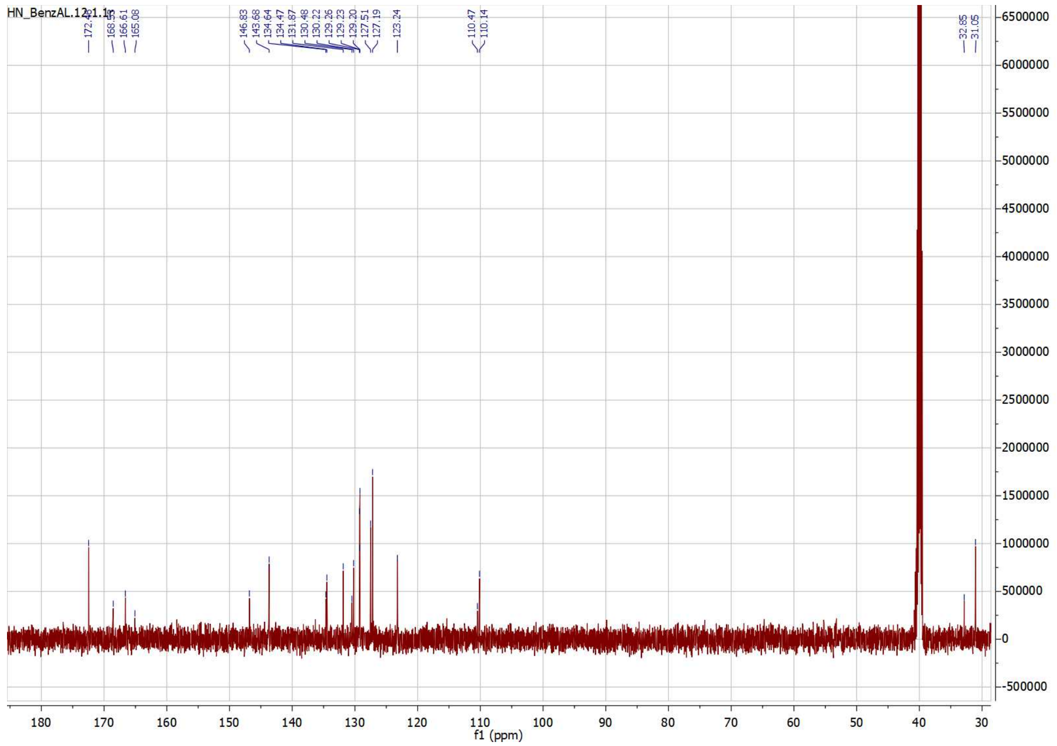

Figure S6. ^{13}H NMR spectrum of **3a** (151 MHz, DMSO-d$_6$).

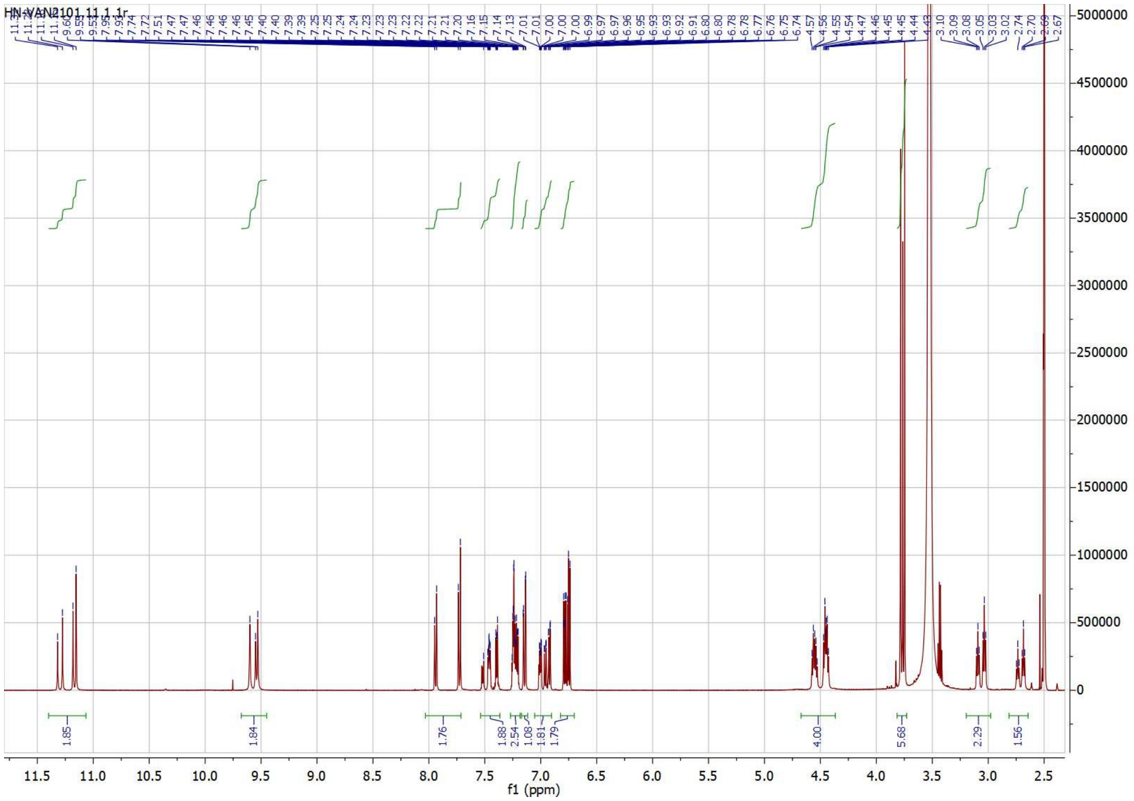

Figure S7. ^{1}H NMR spectrum of **3b** (600 MHz, DMSO-d_6).

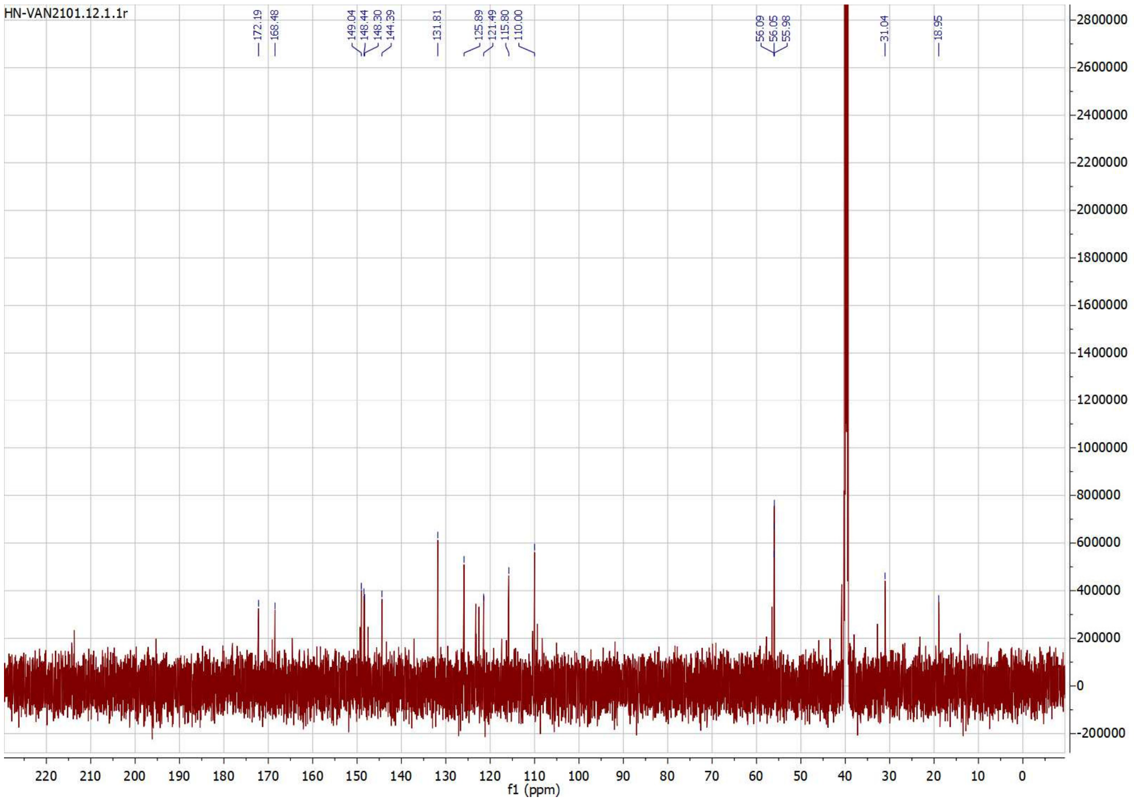

Figure S8. ^{13}H NMR spectrum of **3b** (151 MHz, DMSO-d_6).

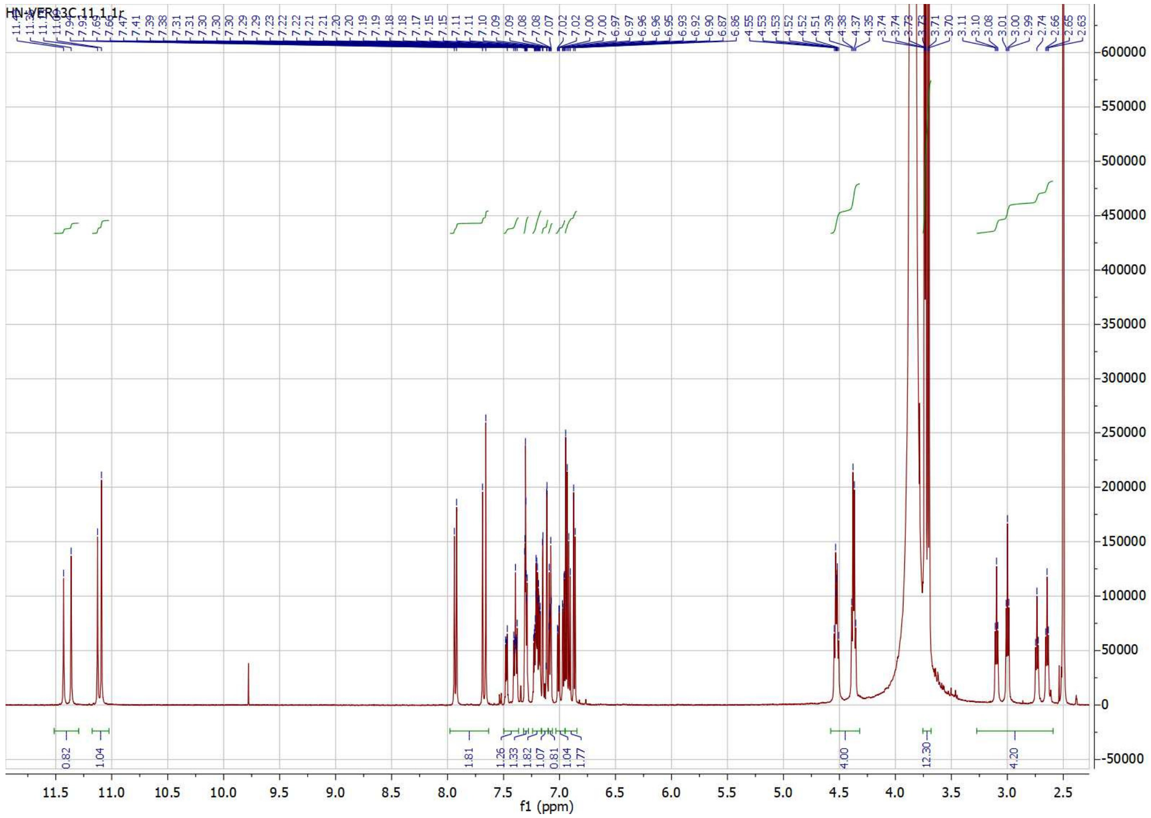

Figure S9. ^{1}H NMR spectrum of **3c** (600 MHz, DMSO-d$_6$)

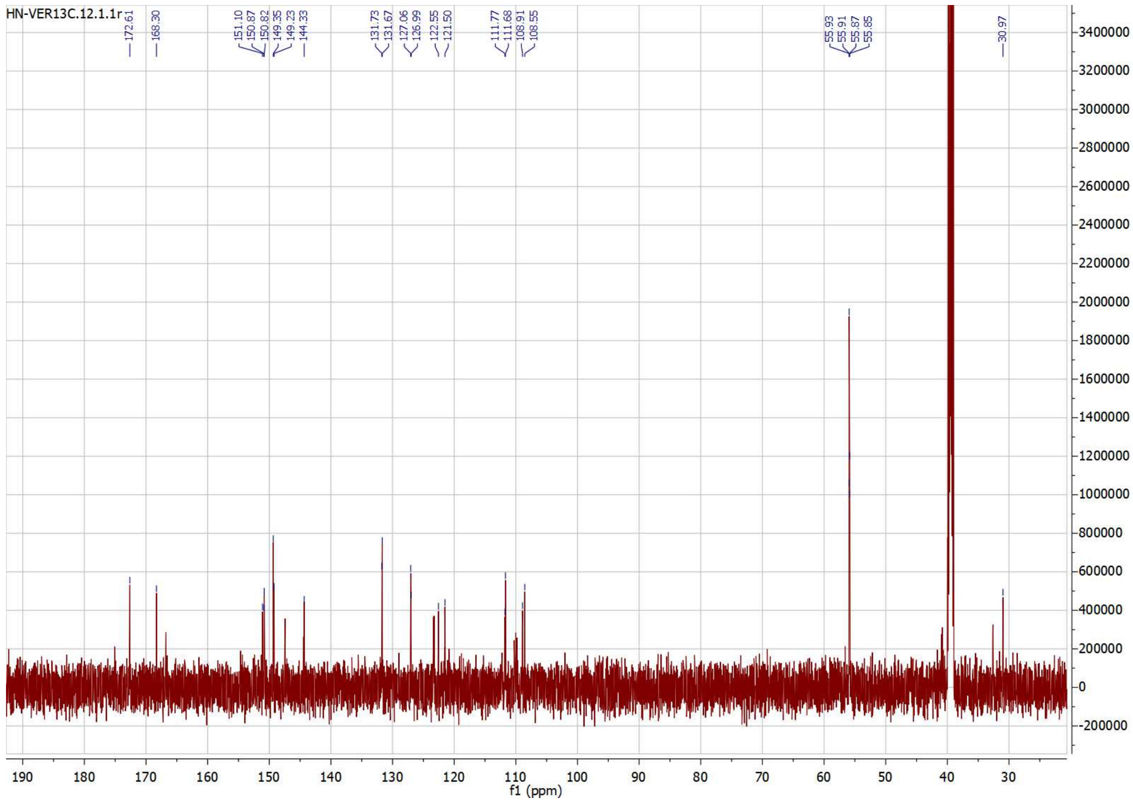

Figure S10. ^{13}H NMR spectrum of **3c** (151 MHz, DMSO-d$_6$).

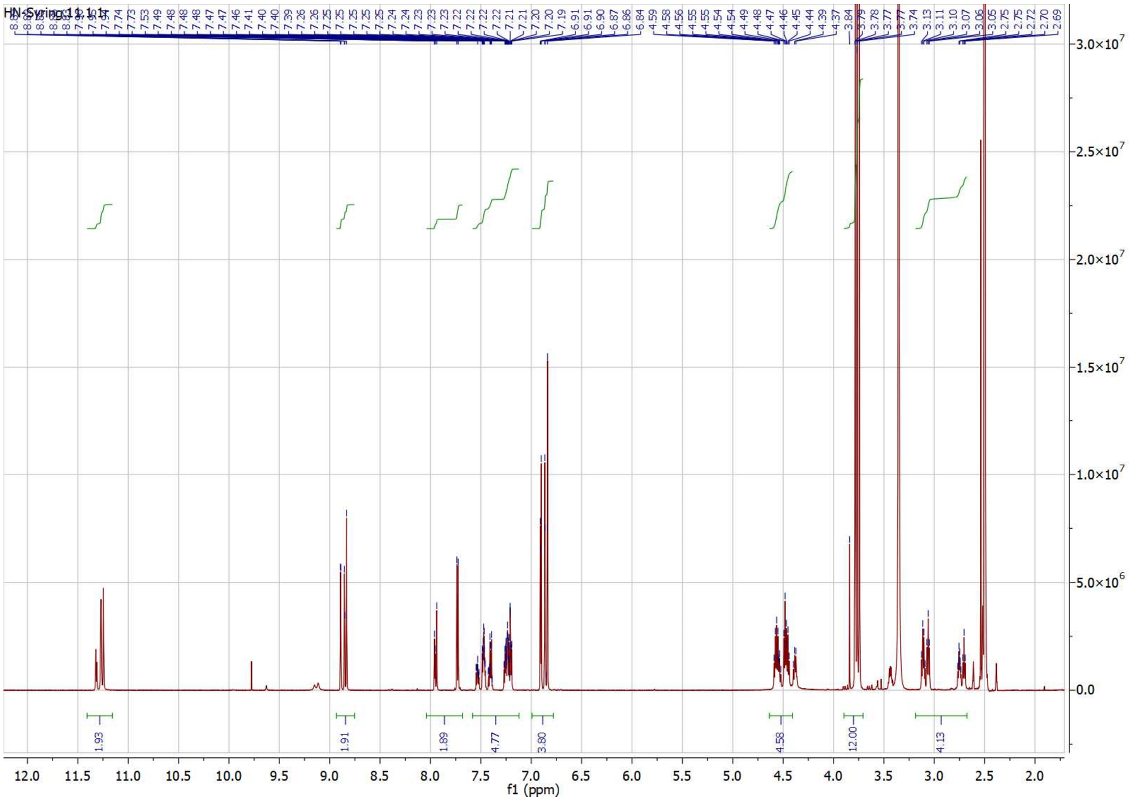

Figure S11. ^{1}H NMR spectrum of **3d** (600 MHz, DMSO-d_6)

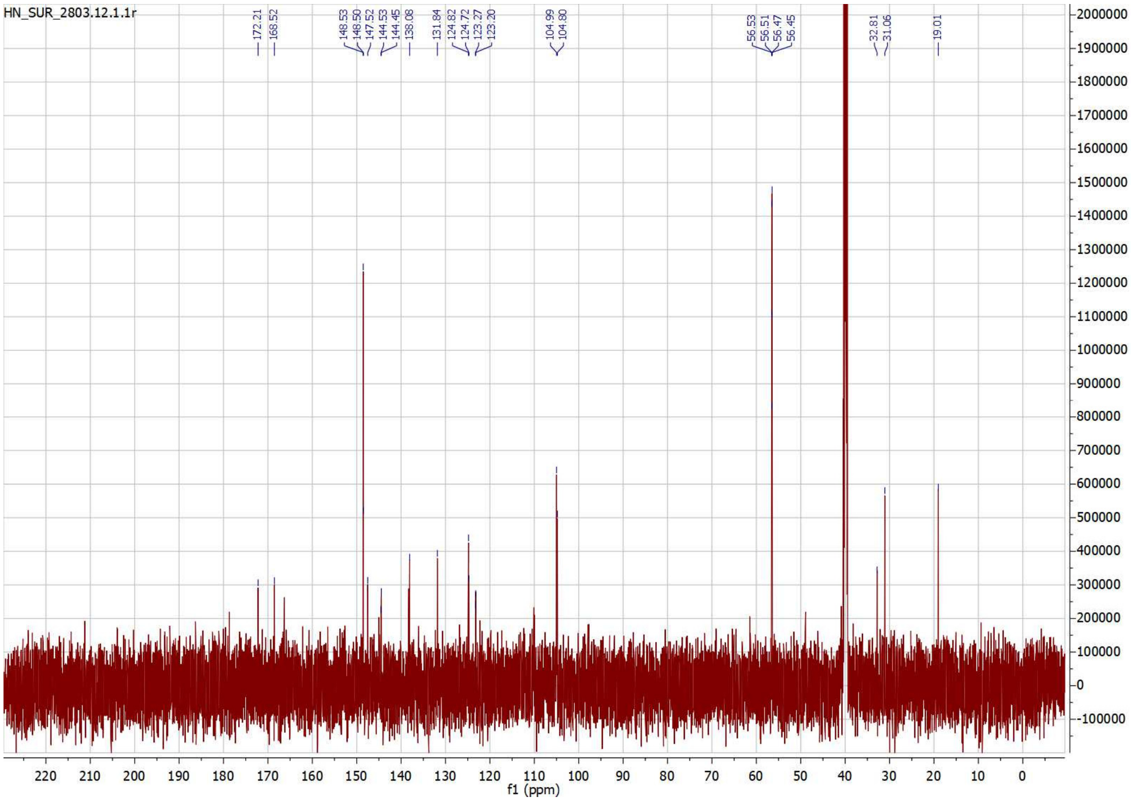

Figure S12. ^{13}H NMR spectrum of **3d** (151 MHz, DMSO-d_6)

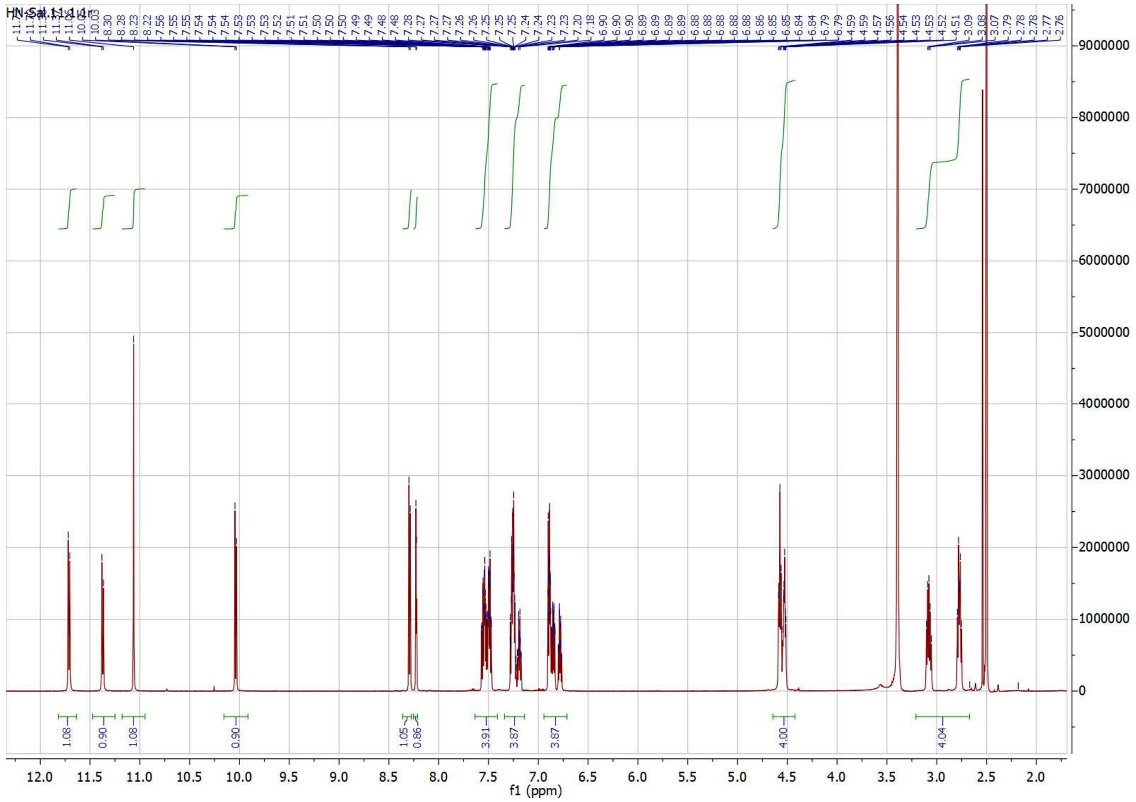

Figure S13. ^{1}H NMR spectrum of **3e** (600 MHz, DMSO-d$_6$)

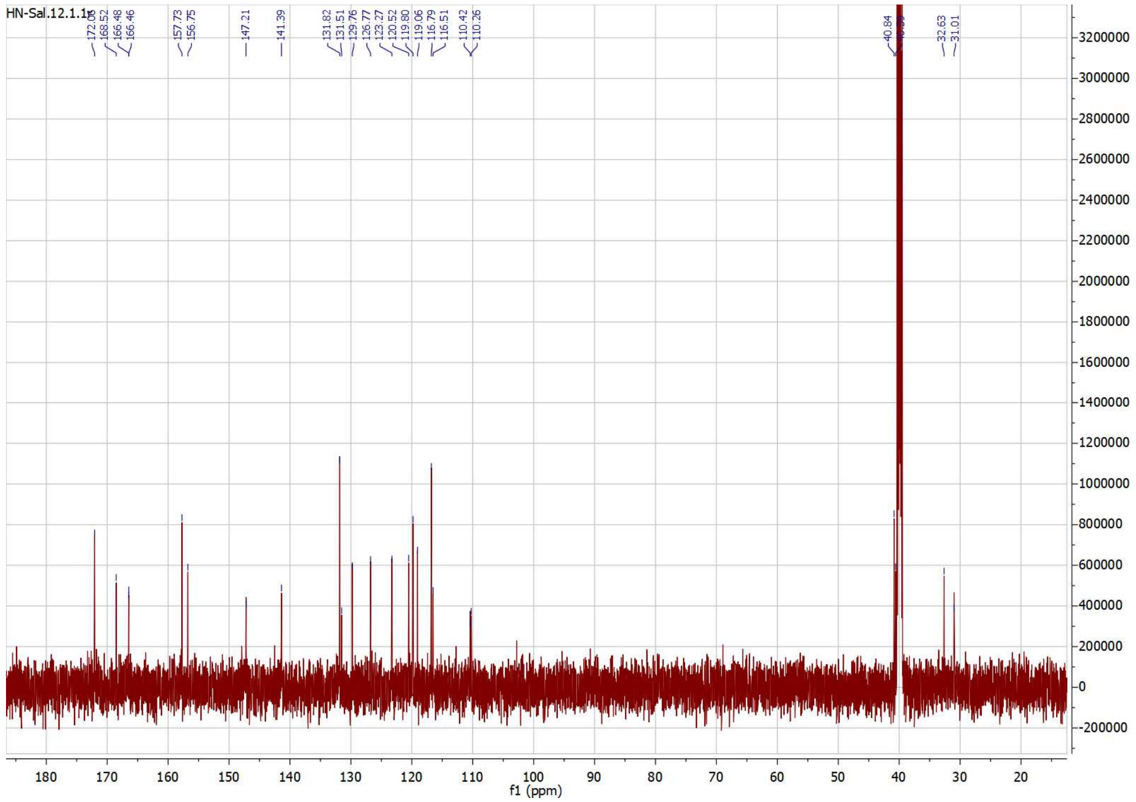

Figure S14. ^{13}H NMR spectrum of **3e** (151 MHz, DMSO-d$_6$)

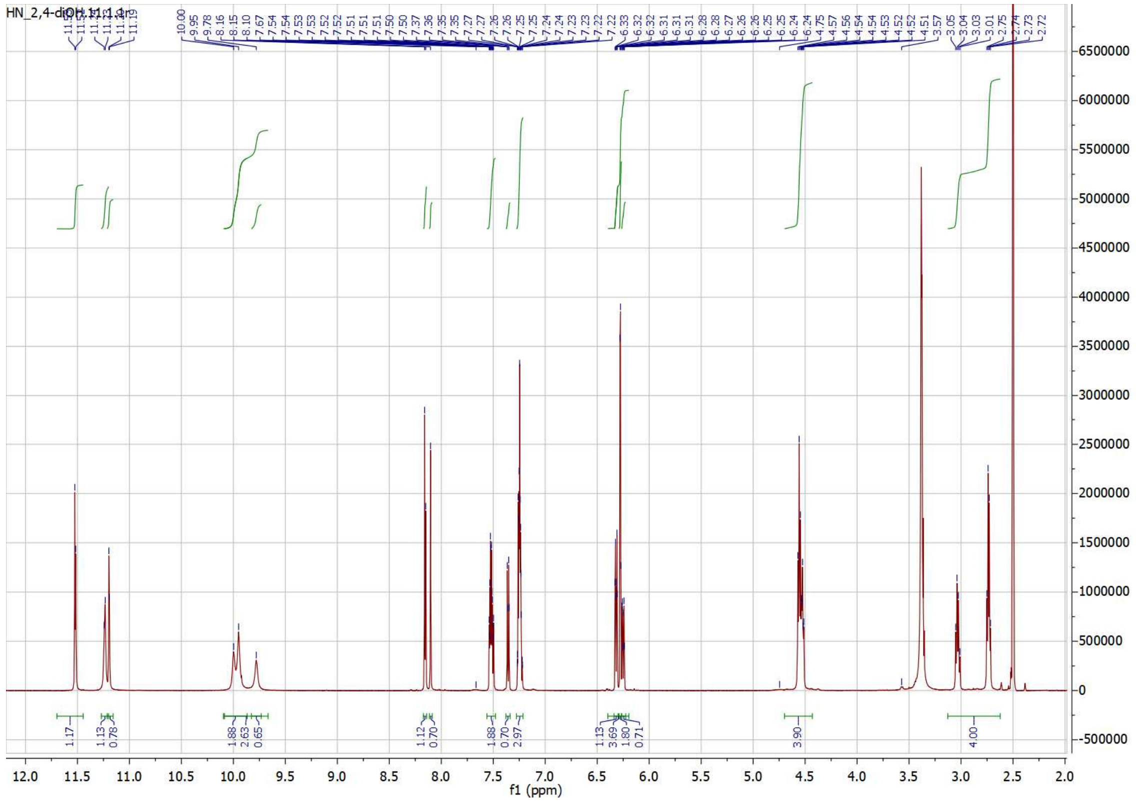

Figure S15. ^{1}H NMR spectrum of **3f** (600 MHz, DMSO-d_6)

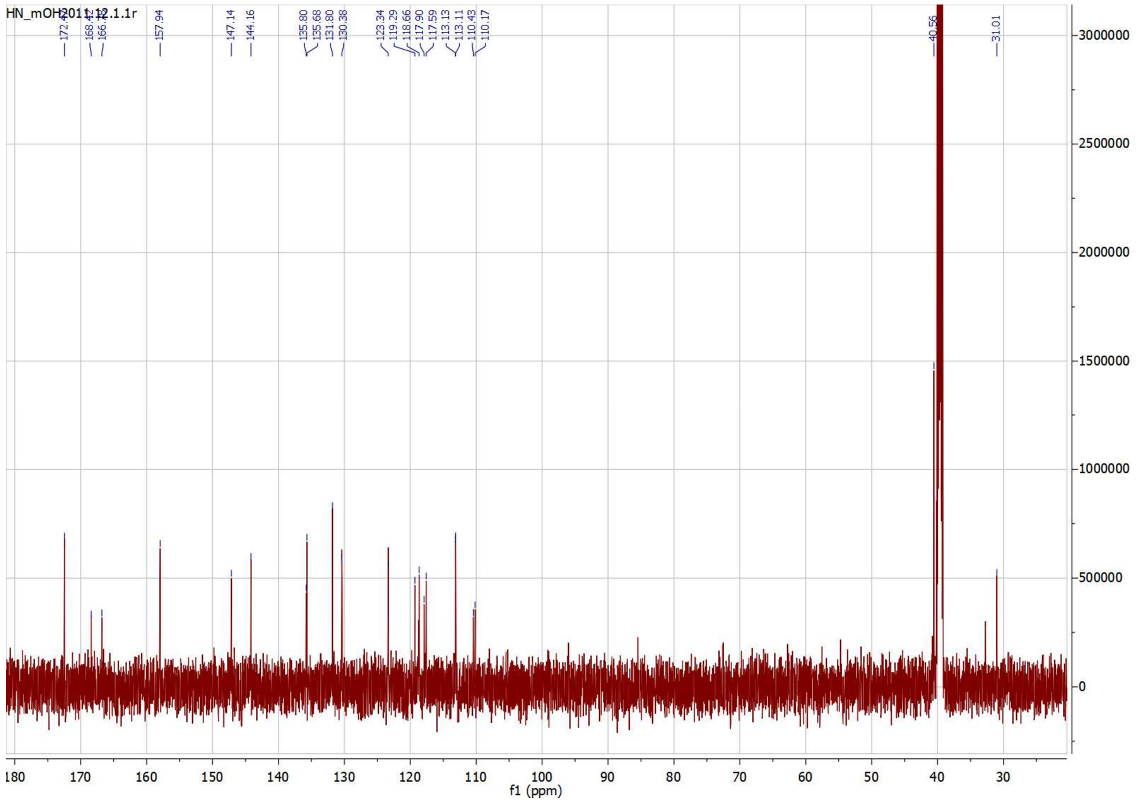

Figure S16. ^{13}H NMR spectrum of **3f** (151 MHz, DMSO-d_6)

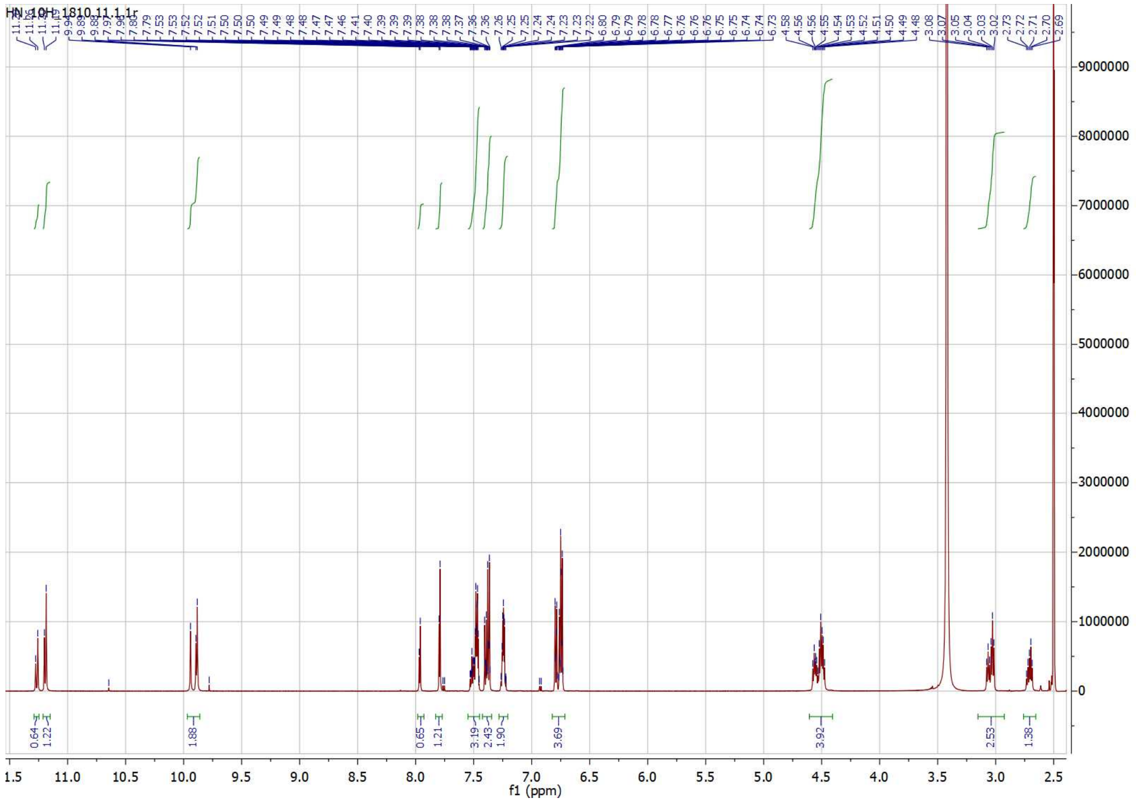

Figure S17. ^{1}H NMR spectrum of **3g** (600 MHz, DMSO-d$_6$)

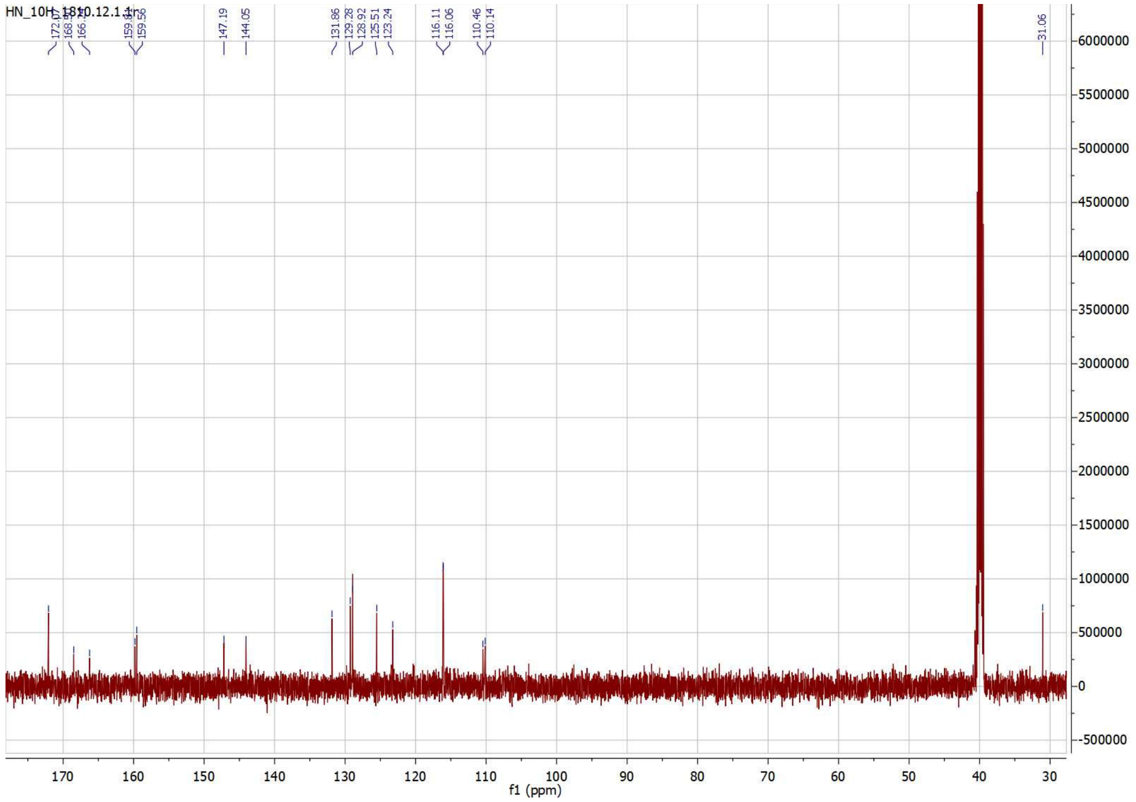

Figure S18. ^{13}H NMR spectrum of **3g** (151 MHz, DMSO-d$_6$)

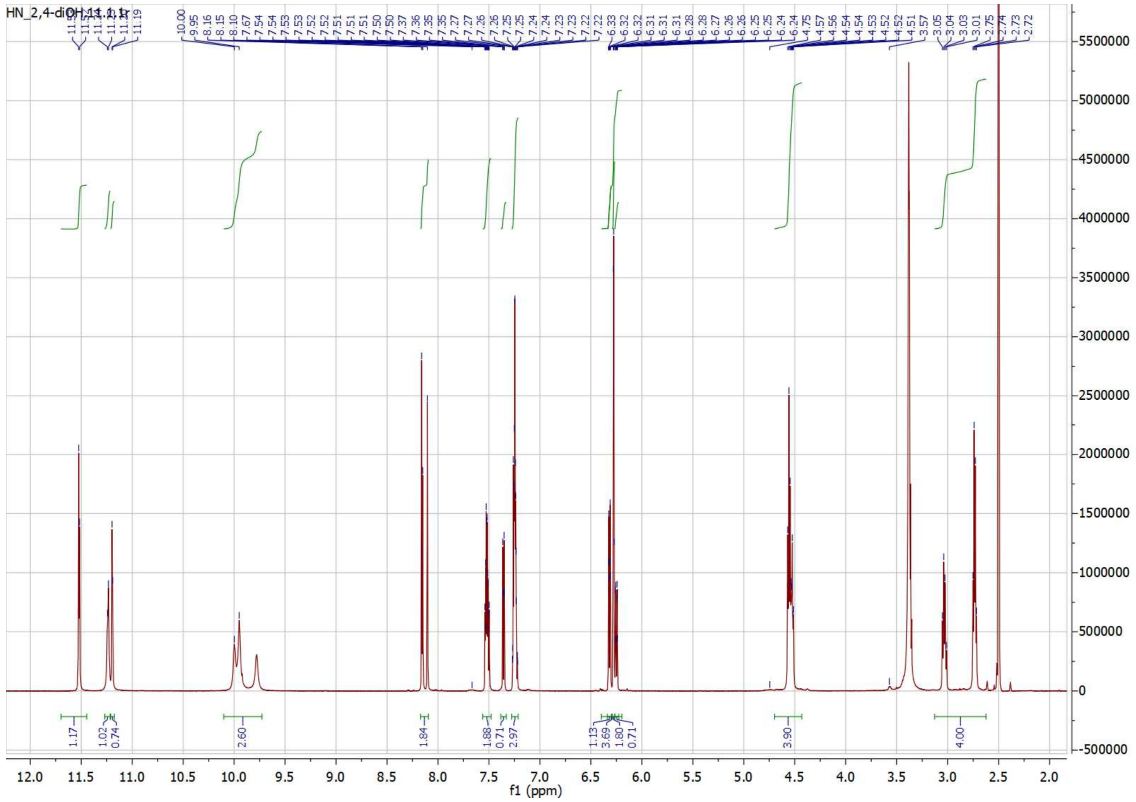

Figure S19. ^{1}H NMR spectrum of **3i** (600 MHz, DMSO-d$_6$)

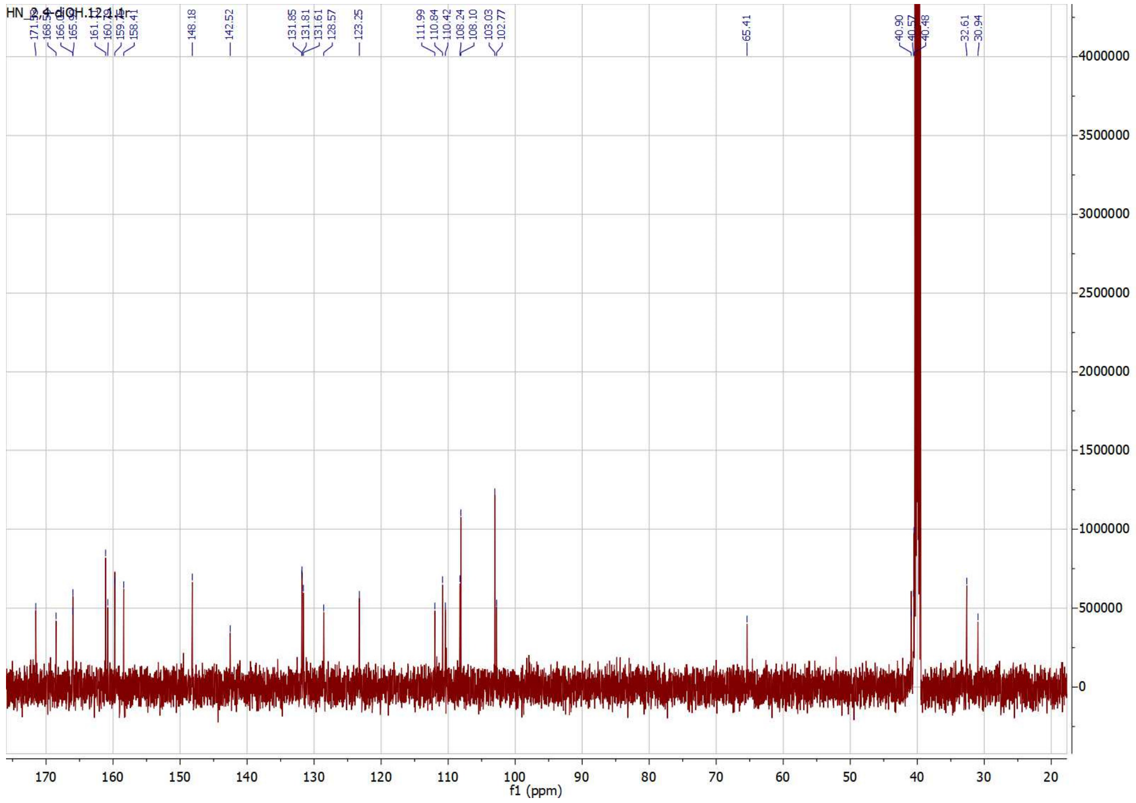

Figure S20. ^{13}H NMR spectrum of **3i** (151 MHz, DMSO-d$_6$)

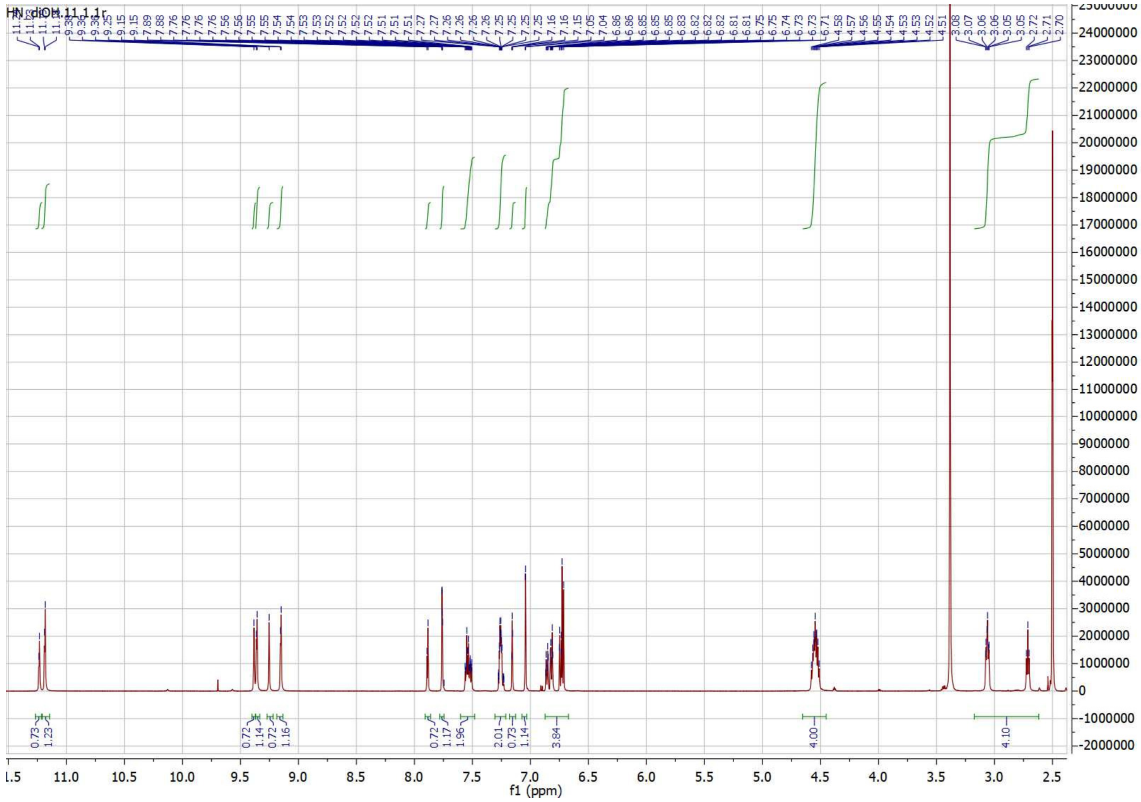

Figure S21. ^{1}H NMR spectrum of **3j** (600 MHz, DMSO-d_6)

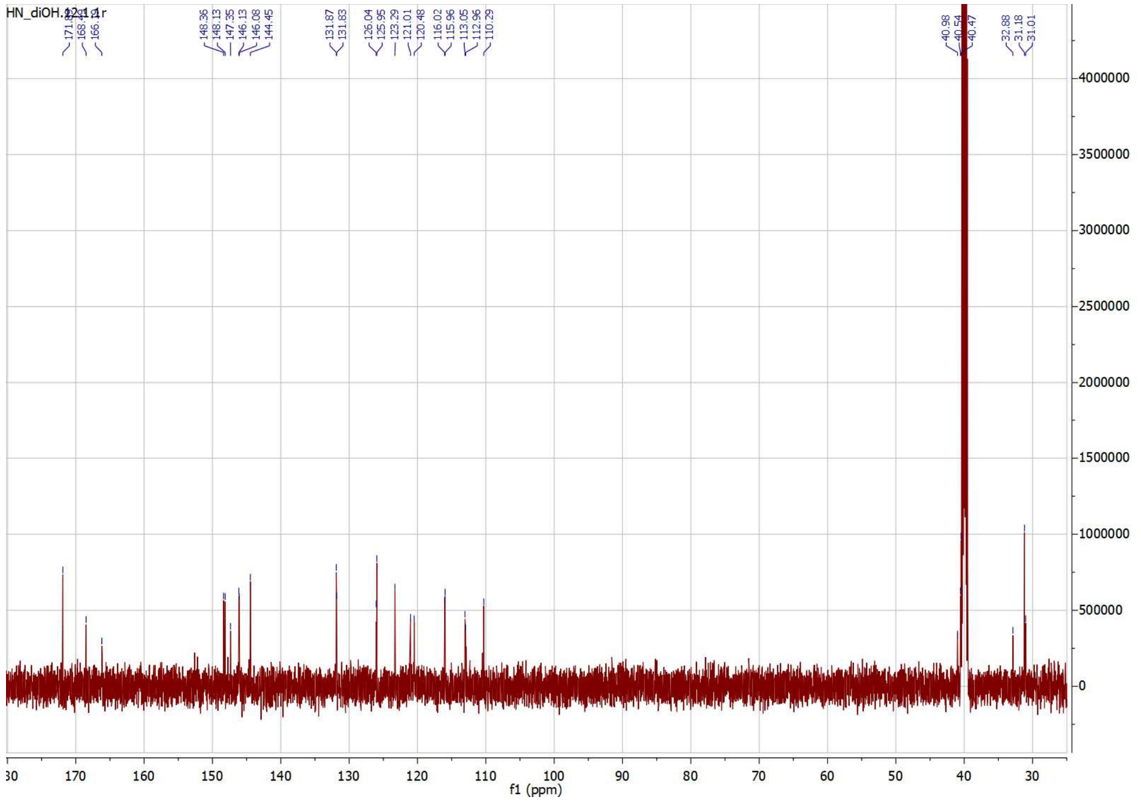

Figure S22. ^{13}H NMR spectrum of **3j** (151 MHz, DMSO-d_6)

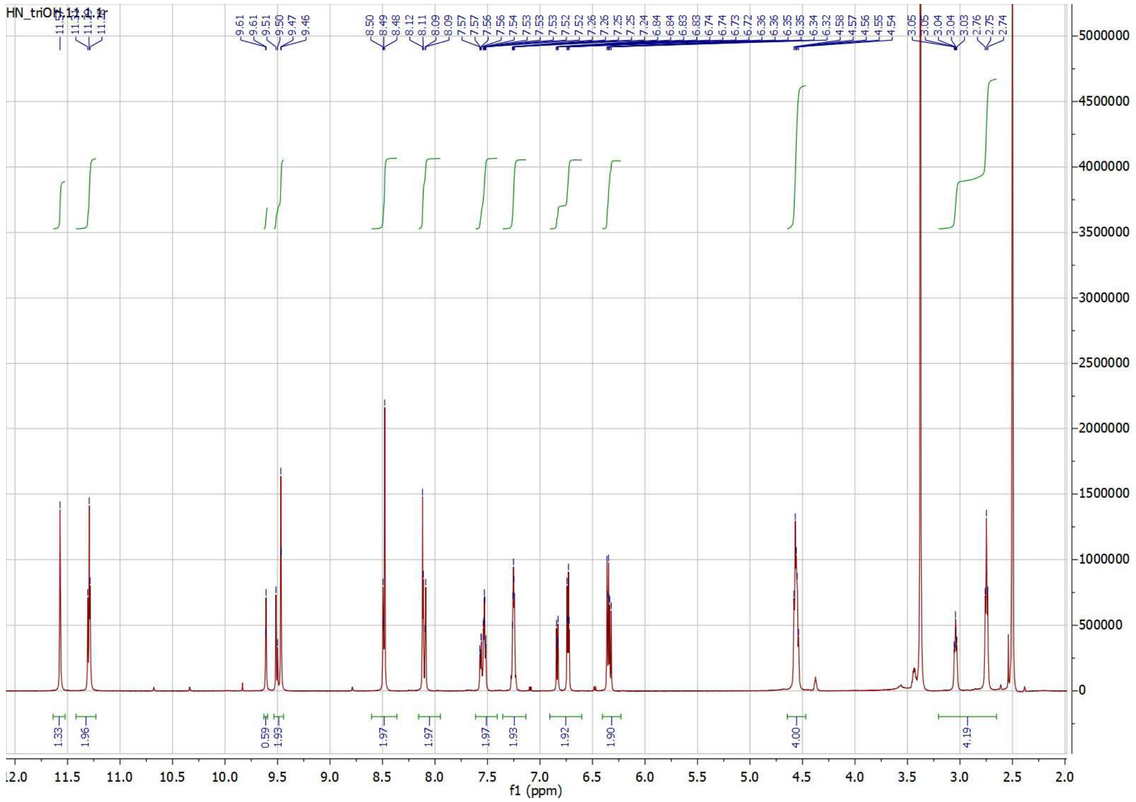

Figure S23. ^{1}H NMR spectrum of **3k** (600 MHz, DMSO-d$_6$)

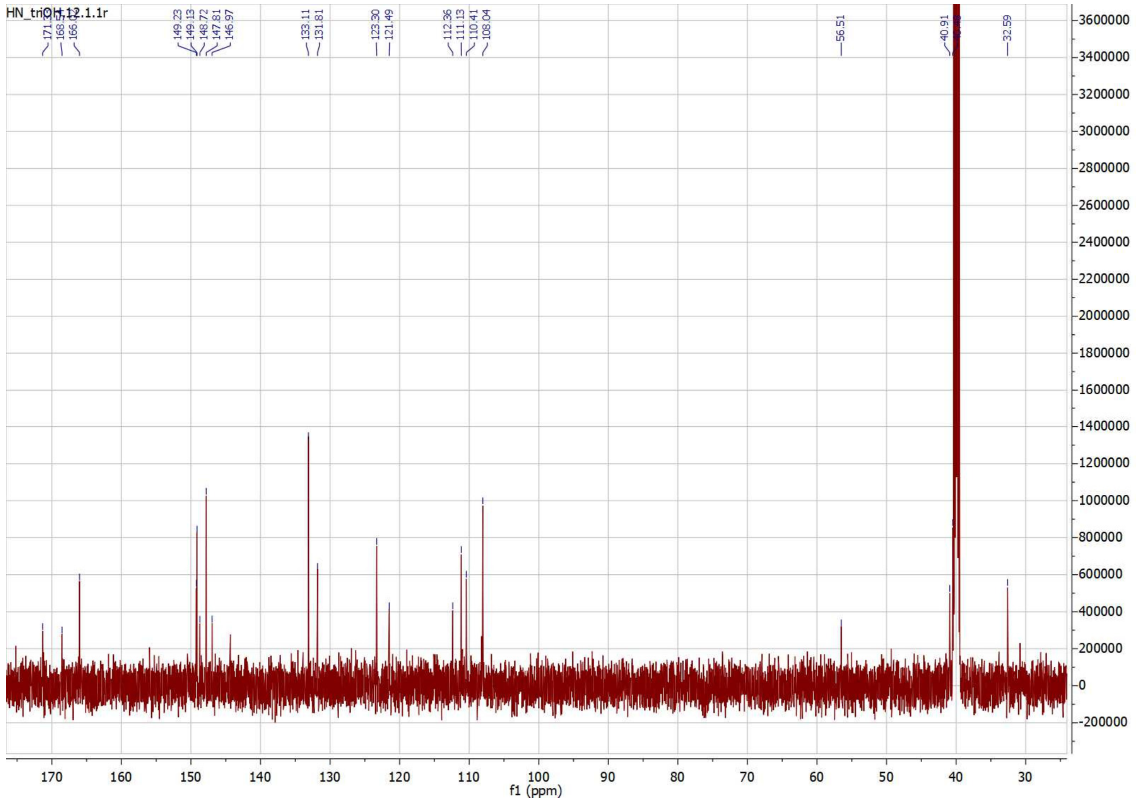

Figure S24. ^{13}H NMR spectrum of **3k** (151 MHz, DMSO-d$_6$)

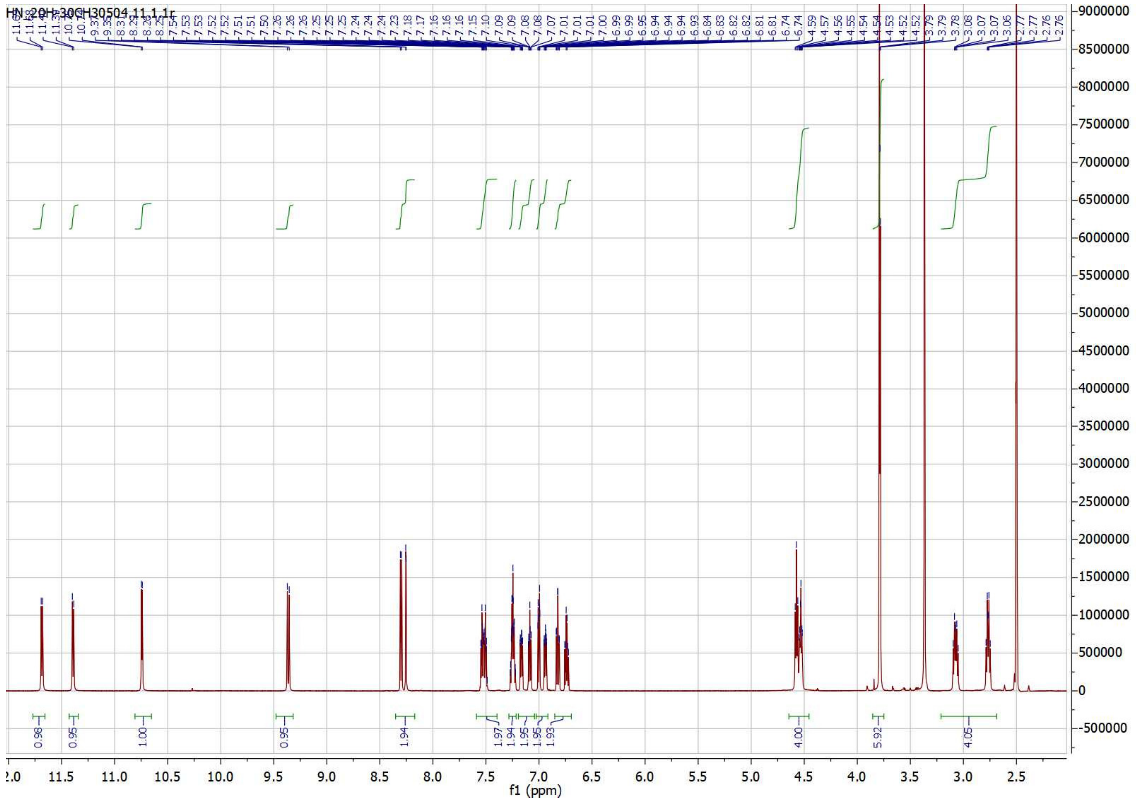

Figure S25. ^{1}H NMR spectrum of **3l** (600 MHz, DMSO-d_6)

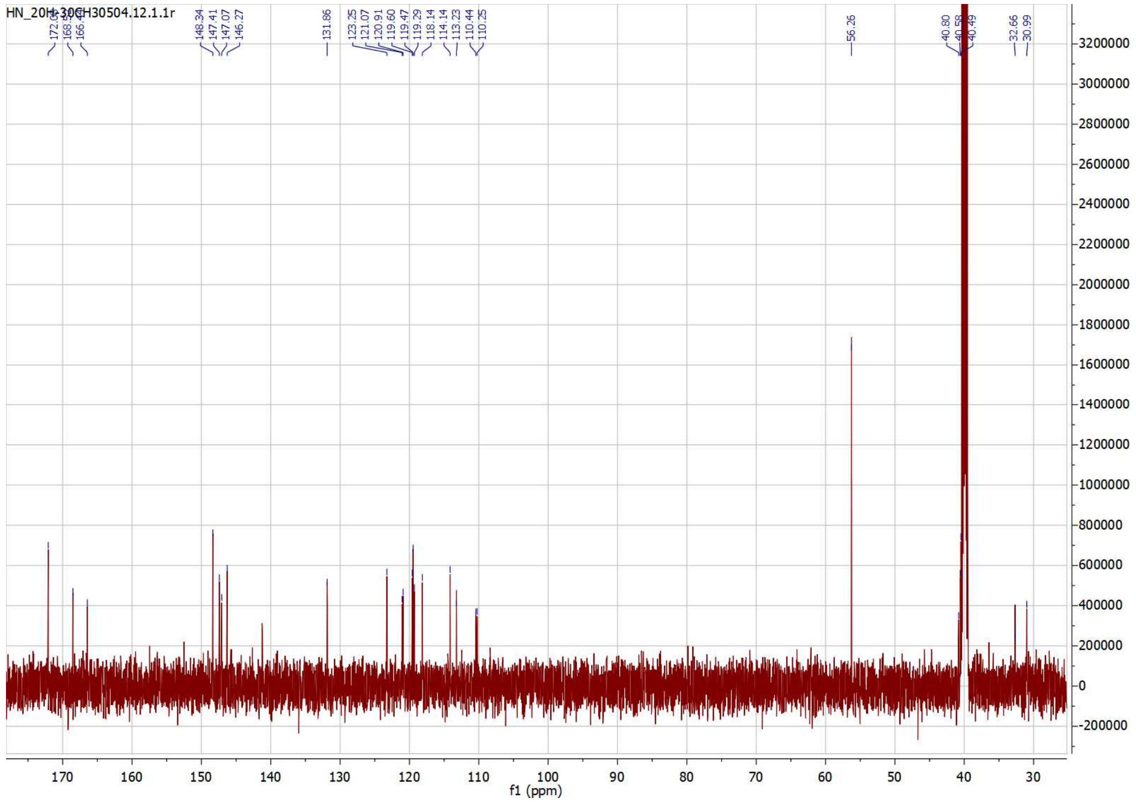

Figure S26. ^{13}H NMR spectrum of **3l** (151 MHz, DMSO-d_6)

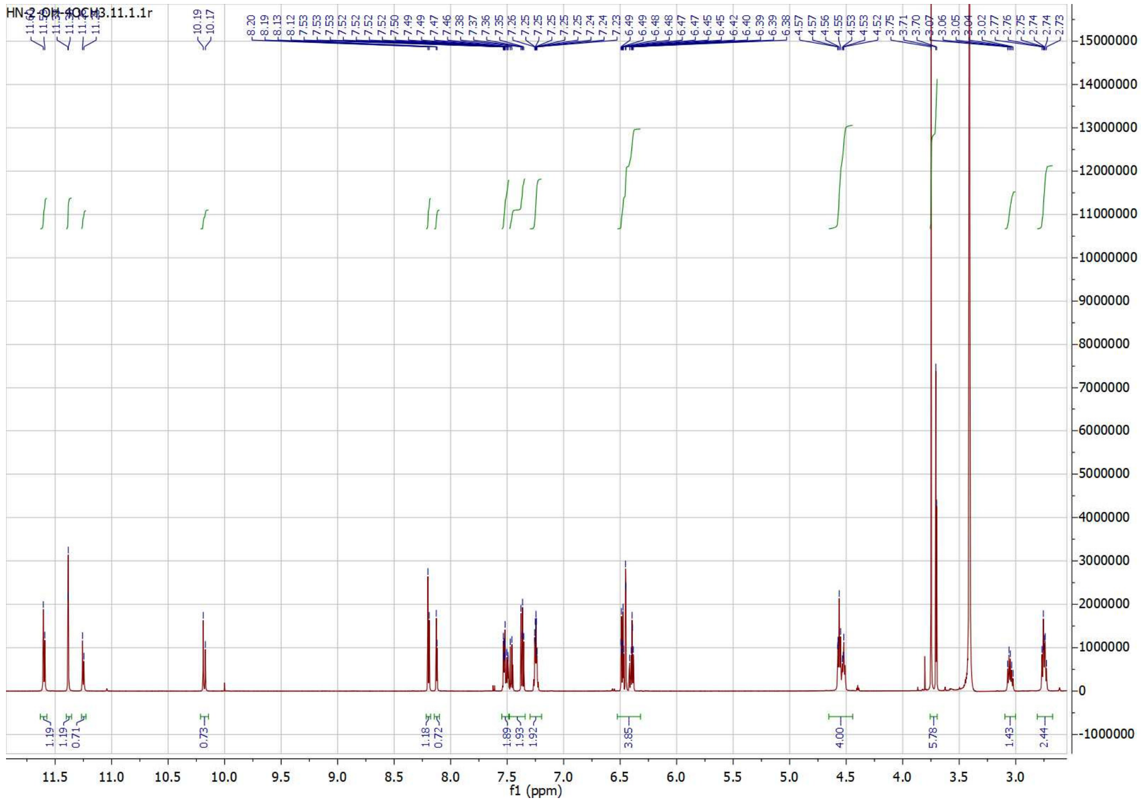

Figure S27. ^{1}H NMR spectrum of **3m** (600 MHz, DMSO-d_6)

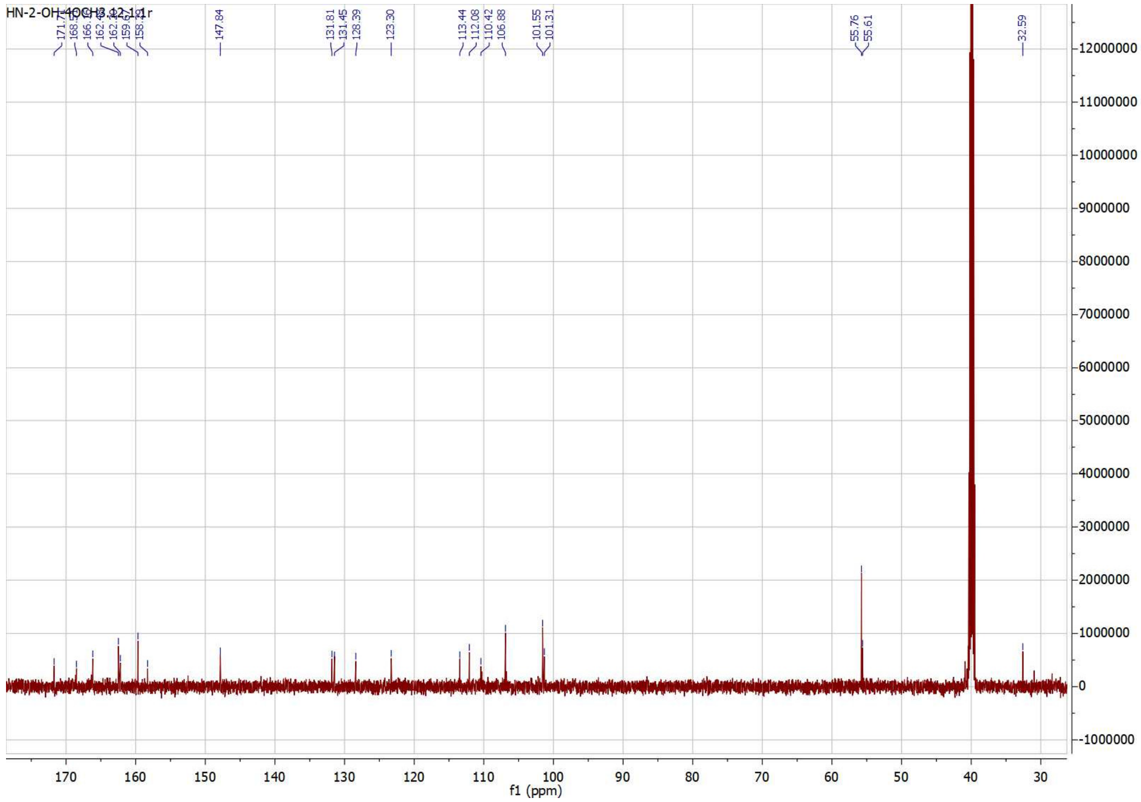

Figure S28. ^{13}H NMR spectrum of **3m** (151 MHz, DMSO-d_6)

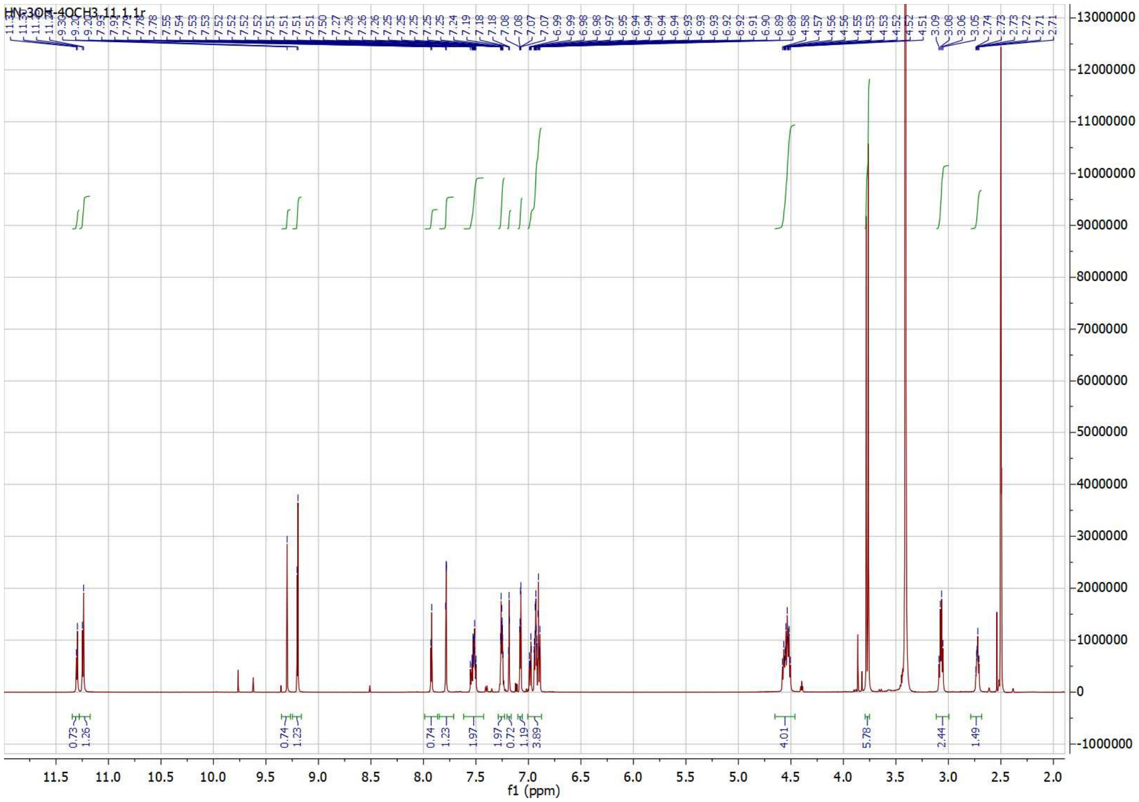

Figure S29. ^{1}H NMR spectrum of **3o** (600 MHz, DMSO-d$_6$)

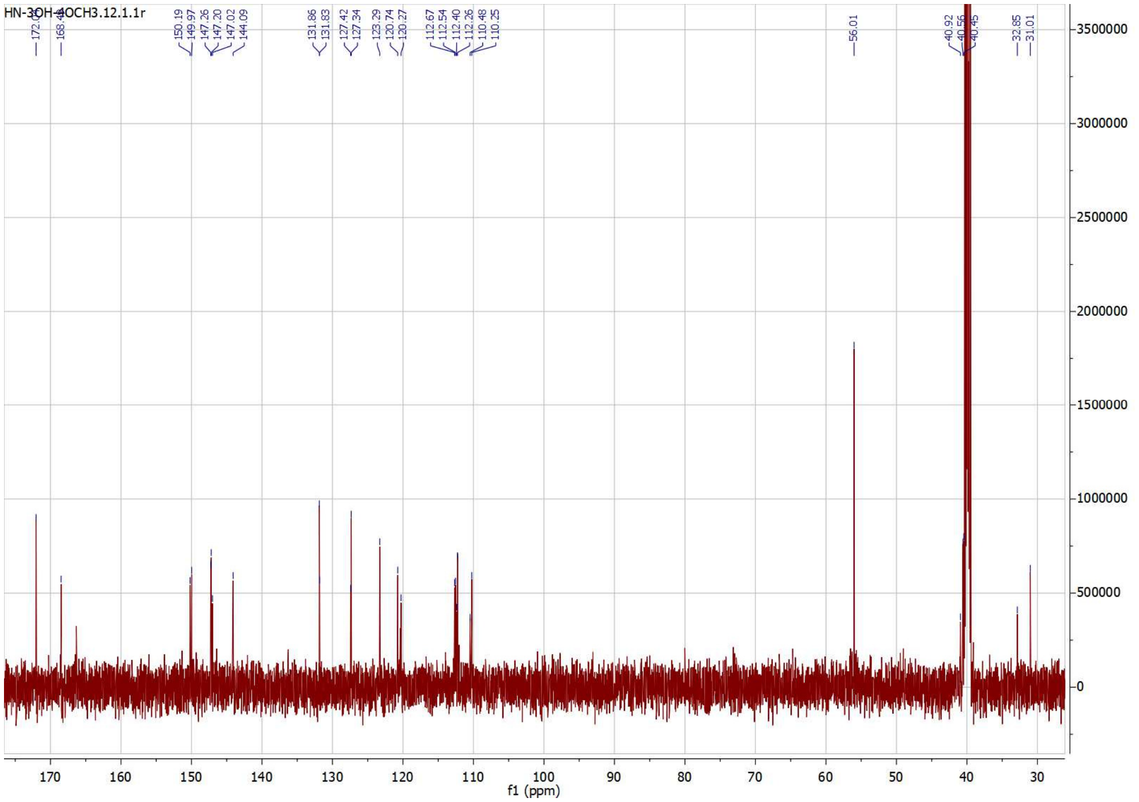

Figure S30. ^{13}H NMR spectrum of **3o** (151 MHz, DMSO-d$_6$)

3. IR spectra of the synthesized compounds

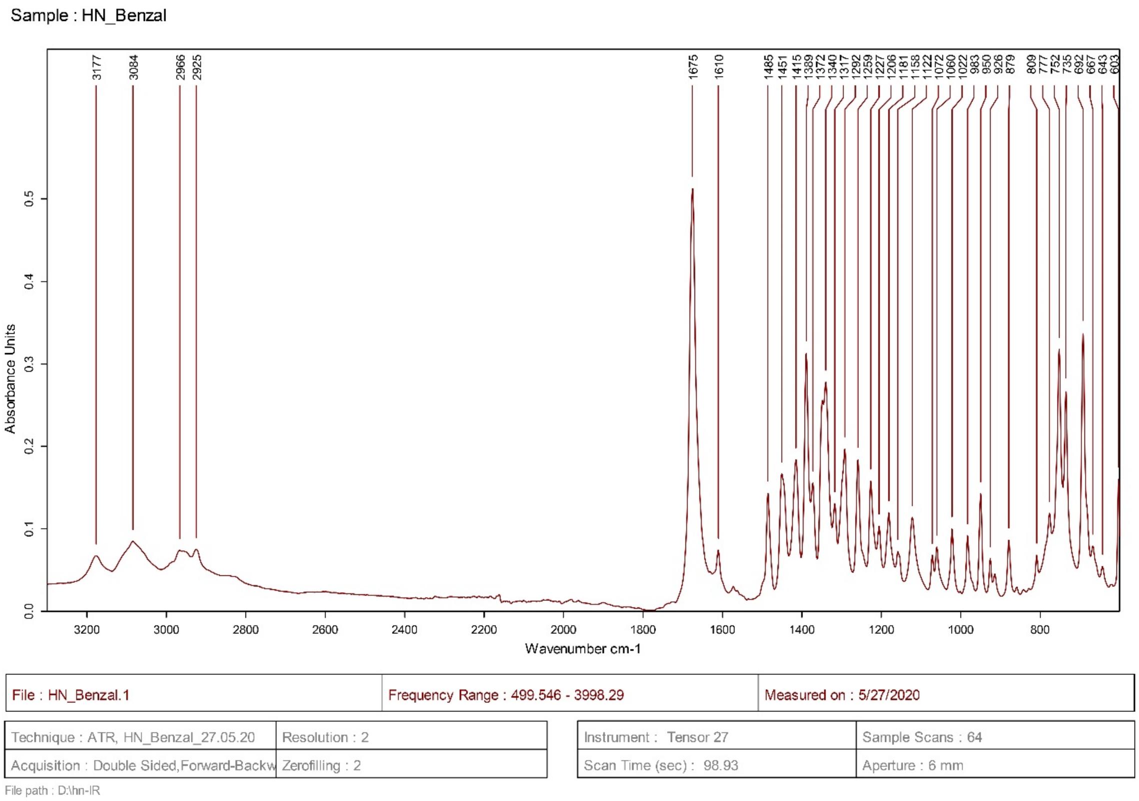

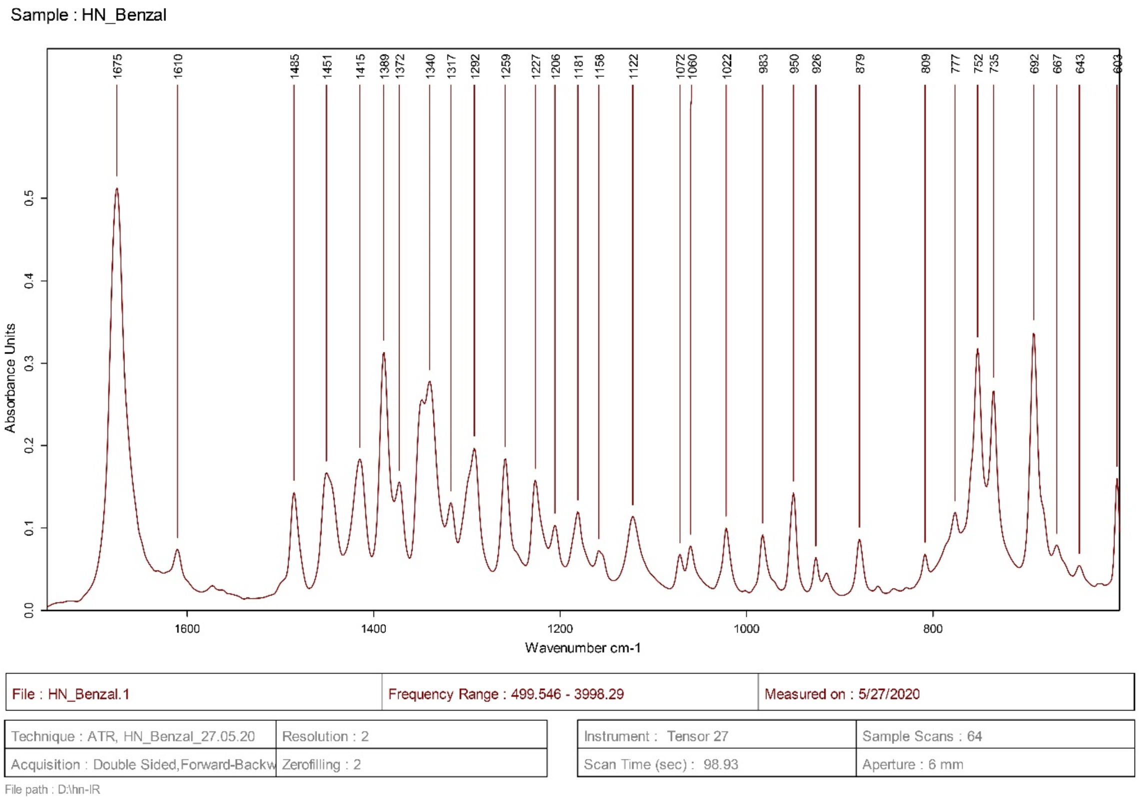

Figure S31. ATR-IR spectrum of **3a**

Sample : HN_Van_0412

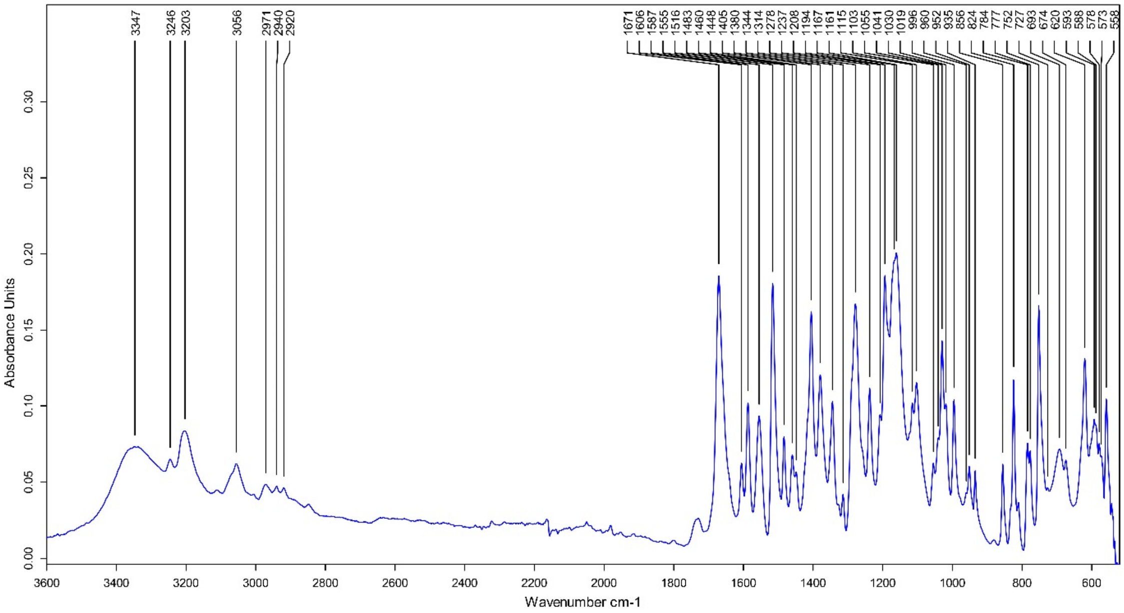

File : HN_Van_0412.0	Frequency Range : 499.546 - 3998.29	Measured on : 12/4/2018

Technique : ATR	Resolution : 2	Instrument : Tensor 27	Sample Scans : 64
Acquisition : Double Sided,Forward-Backw	Zerofilling : 2	Scan Time (sec) : 98.974	Aperture : 6 mm

File path : C:\Users\Public\Documents\Bruker\OPUS_8.7.10\MEAS\Tenzor_27_Archiv\Service\Neda

Sample : HN_Van_0412

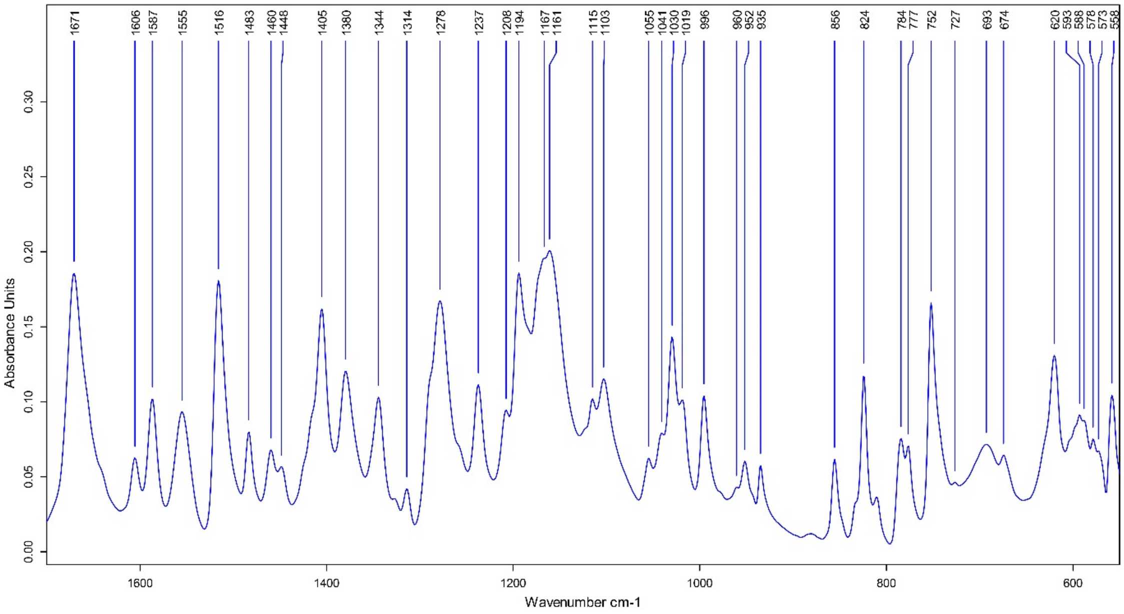

File : HN_Van_0412.0	Frequency Range : 499.546 - 3998.29	Measured on : 12/4/2018

Technique : ATR	Resolution : 2	Instrument : Tensor 27	Sample Scans : 64
Acquisition : Double Sided,Forward-Backw	Zerofilling : 2	Scan Time (sec) : 98.974	Aperture : 6 mm

File path : C:\Users\Public\Documents\Bruker\OPUS_8.7.10\MEAS\Tenzor_27_Archiv\Service\Neda

Figure S32. ATR-IR spectrum of **3b**

Sample : HN_Veratral

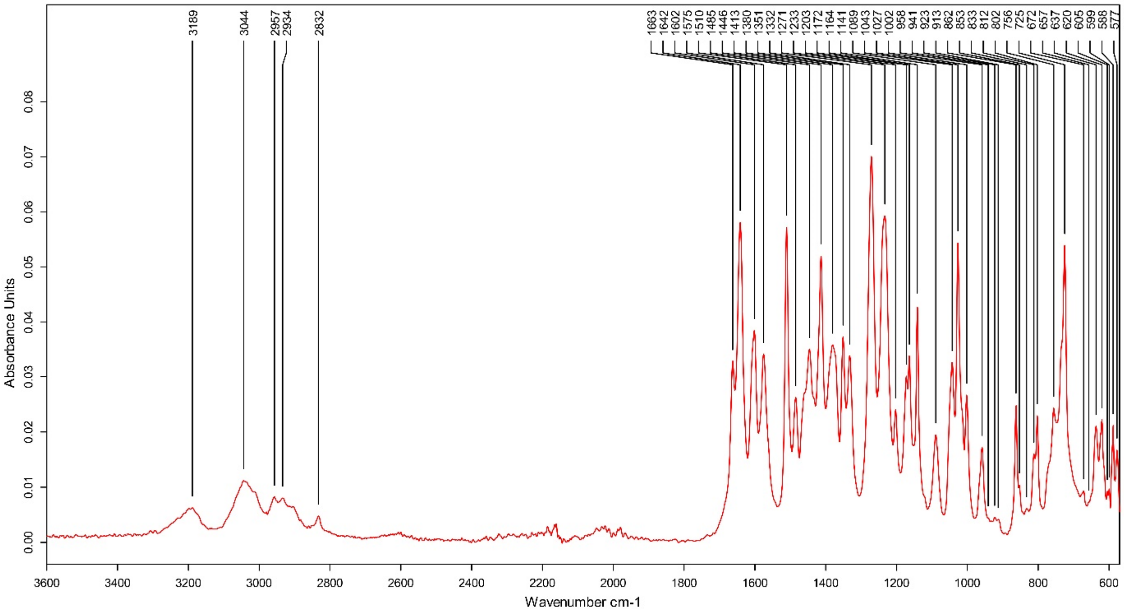

File : HN_Veratral.0	Frequency Range : 499.528 - 3998.15	Measured on : 2/21/2018

Technique : ATR, 64 scans; 2 cm-1 resolu	Resolution : 2	Instrument : Tensor 27	Sample Scans : 64
Acquisition : Double Sided,Forward-Backw	Zerofilling : 2	Scan Time (sec) : 98.9399	Aperture : 3.5 mm

File path : C:\Users\Public\Documents\Bruker\OPUS_8.7.10\MEAS\Tenzor_27_Archiv\Service\Neda

Sample : HN_Veratral

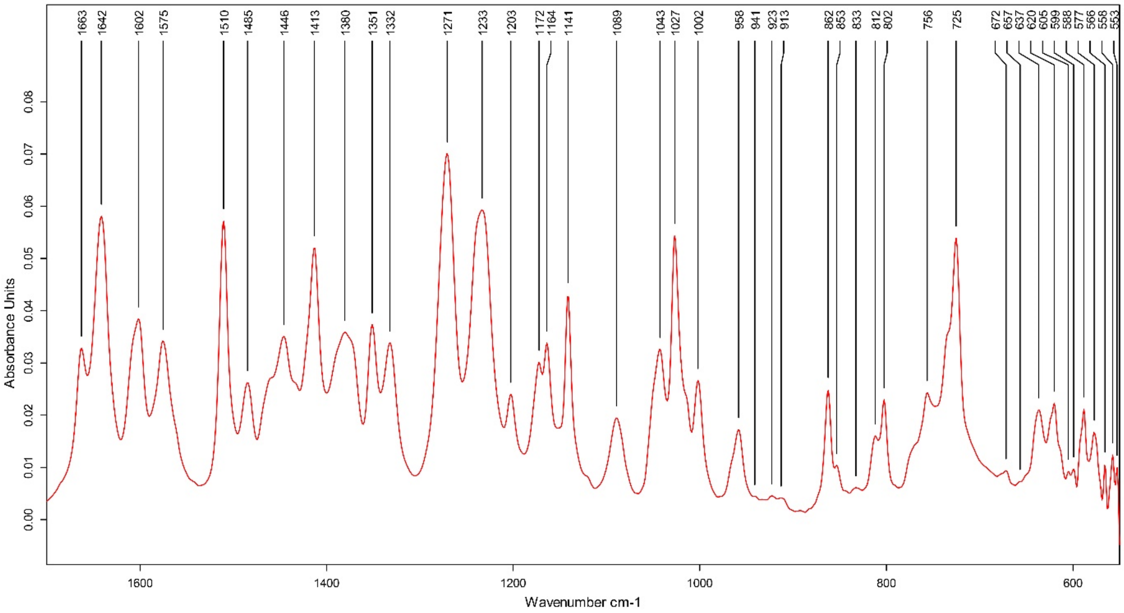

File : HN_Veratral.0	Frequency Range : 499.528 - 3998.15	Measured on : 2/21/2018

Technique : ATR, 64 scans; 2 cm-1 resolu	Resolution : 2	Instrument : Tensor 27	Sample Scans : 64
Acquisition : Double Sided,Forward-Backw	Zerofilling : 2	Scan Time (sec) : 98.9399	Aperture : 3.5 mm

File path : C:\Users\Public\Documents\Bruker\OPUS_8.7.10\MEAS\Tenzor_27_Archiv\Service\Neda

Figure S33. ATR-IR spectrum of **3c**

Sample : HN-Syring-22032019

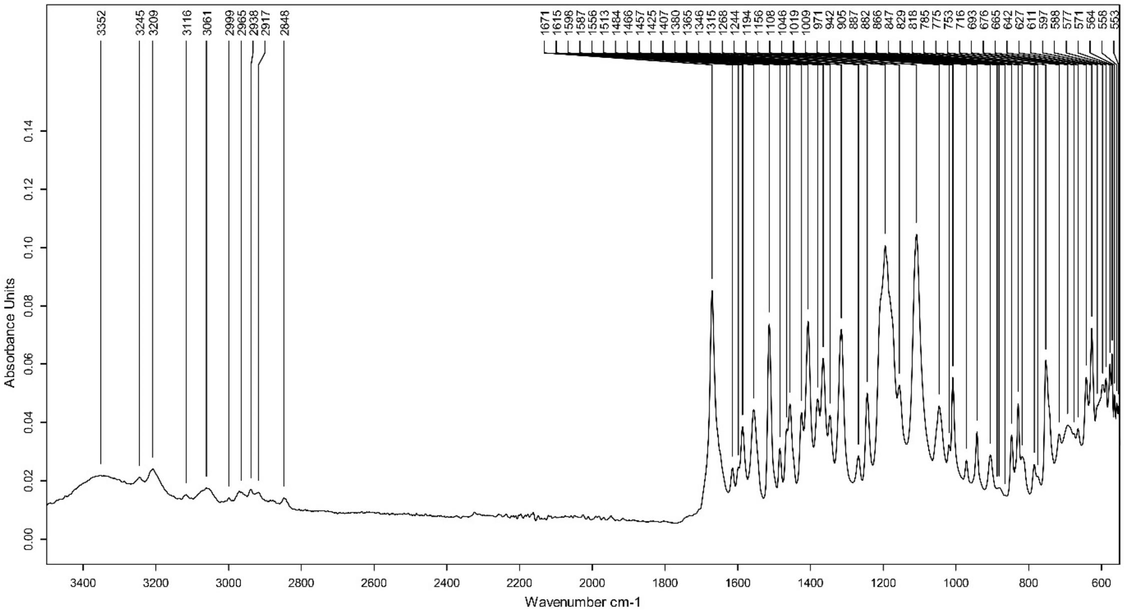

File : HN-Syring-22032019.0 | Frequency Range : 499.546 - 3998.29 | Measured on : 3/22/2019

Technique : ATR, novo polucavane | Resolution : 2 | Instrument : Tensor 27 | Sample Scans : 64

Acquisition : Double Sided,Forward-Backw | Zerofilling : 2 | Scan Time (sec) : 98.941 | Aperture : 6 mm

File path : C:\Users\Public\Documents\Bruker\OPUS_8.7.10\MEAS\Tenzor_27_Archiv\Service\Neda

Sample : HN-Syring-22032019

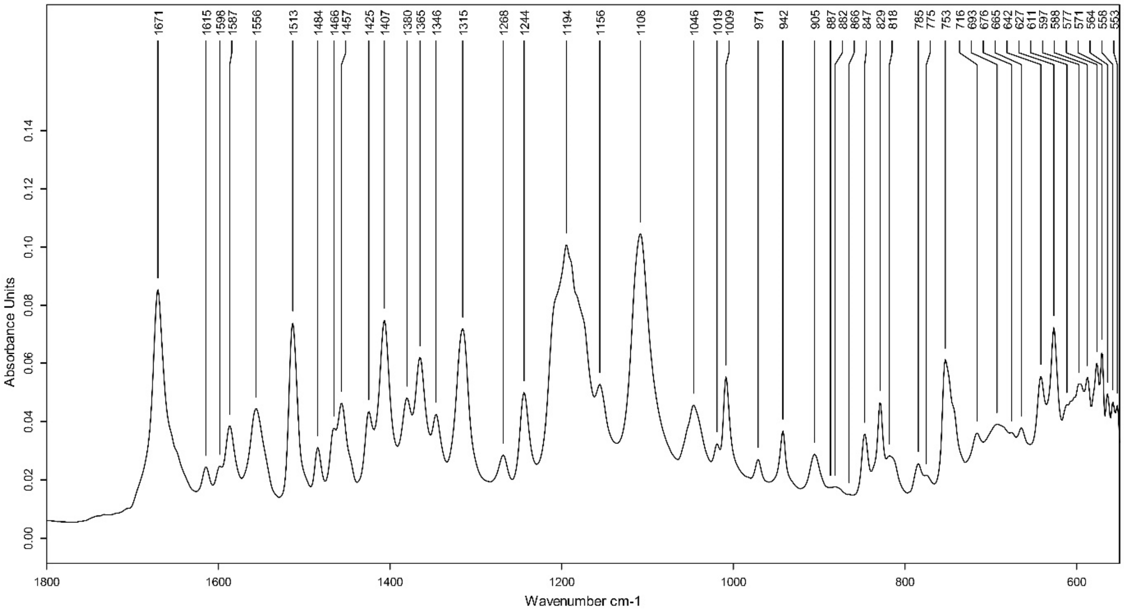

File : HN-Syring-22032019.0 | Frequency Range : 499.546 - 3998.29 | Measured on : 3/22/2019

Technique : ATR, novo polucavane | Resolution : 2 | Instrument : Tensor 27 | Sample Scans : 64

Acquisition : Double Sided,Forward-Backw | Zerofilling : 2 | Scan Time (sec) : 98.941 | Aperture : 6 mm

File path : C:\Users\Public\Documents\Bruker\OPUS_8.7.10\MEAS\Tenzor_27_Archiv\Service\Neda

Figure S34. ATR-IR spectrum of **3d**

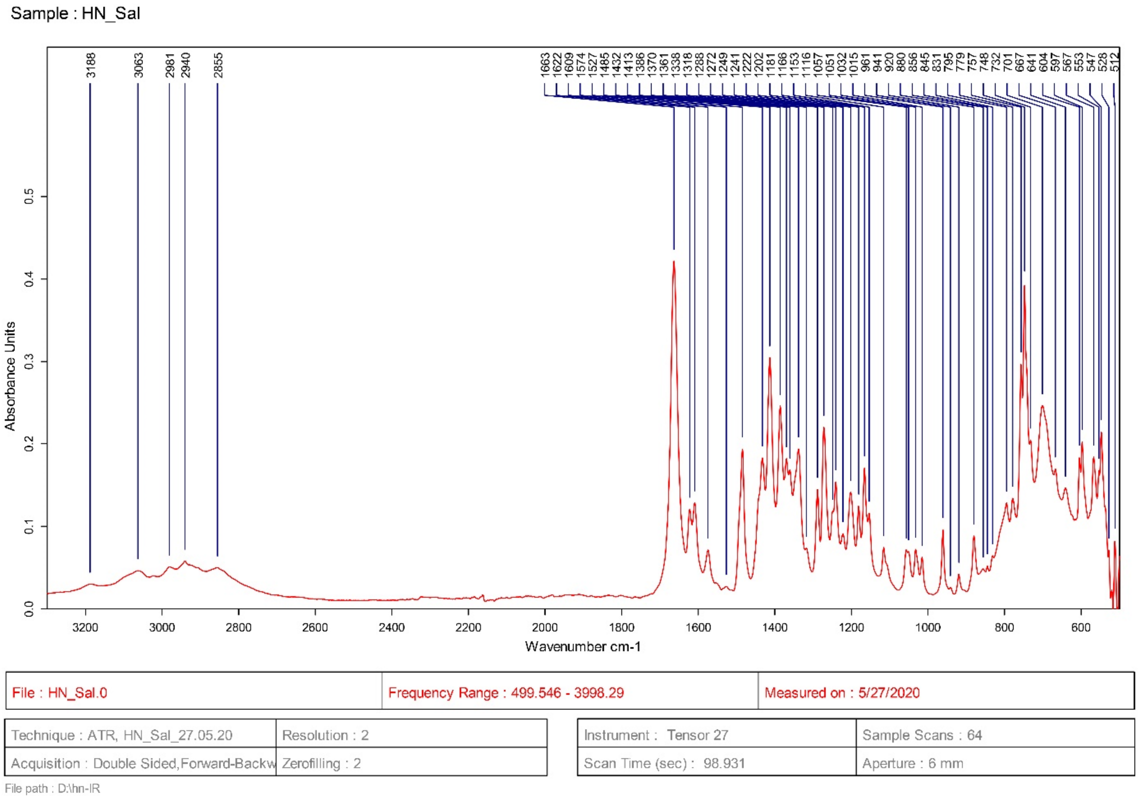

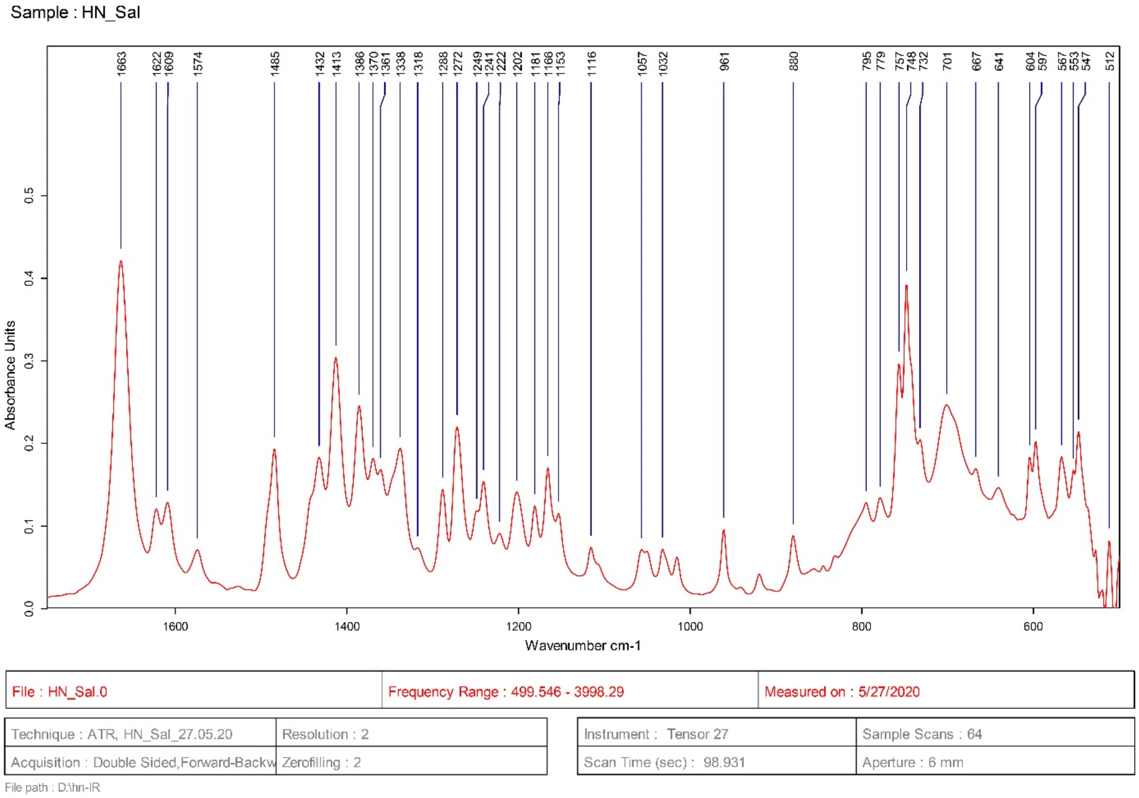

Figure S35. ATR-IR spectrum of **3e**

Sample : HN_m-OH3

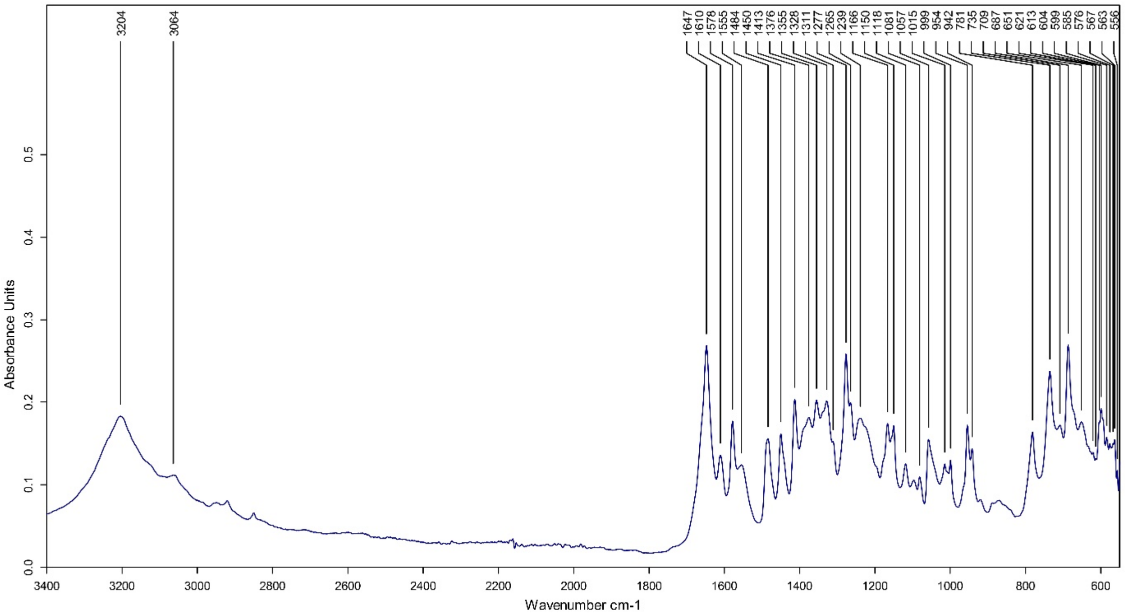

File : HN_m-OH3.0	Frequency Range : 499.546 - 3998.29	Measured on : 10/23/2018

Technique : ATR	Resolution : 2	Instrument : Tensor 27	Sample Scans : 64
Acquisition : Double Sided,Forward-Backw	Zerofilling : 2	Scan Time (sec) : 98.985	Aperture : 4 mm

File path : D:\IR\Neda

Sample : HN_m-OH3

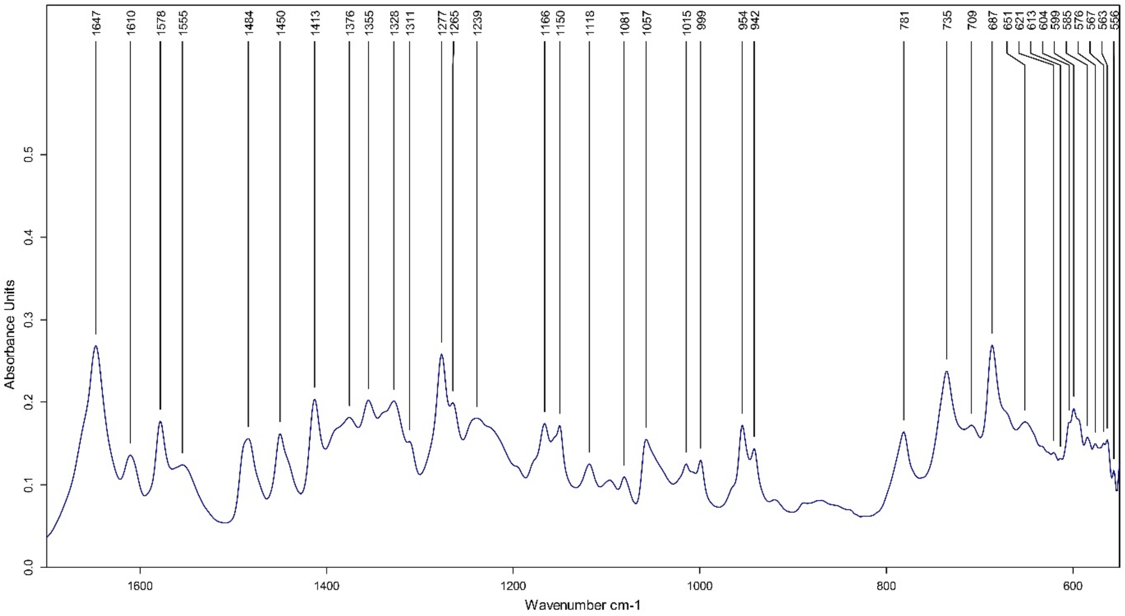

File : HN_m-OH3.0	Frequency Range : 499.546 - 3998.29	Measured on : 10/23/2018

Technique : ATR	Resolution : 2	Instrument : Tensor 27	Sample Scans : 64
Acquisition : Double Sided,Forward-Backw	Zerofilling : 2	Scan Time (sec) : 98.985	Aperture : 4 mm

File path : D:\IR\Neda

Figure S36. ATR-IR spectrum of **3f**

Sample : HN_pOH

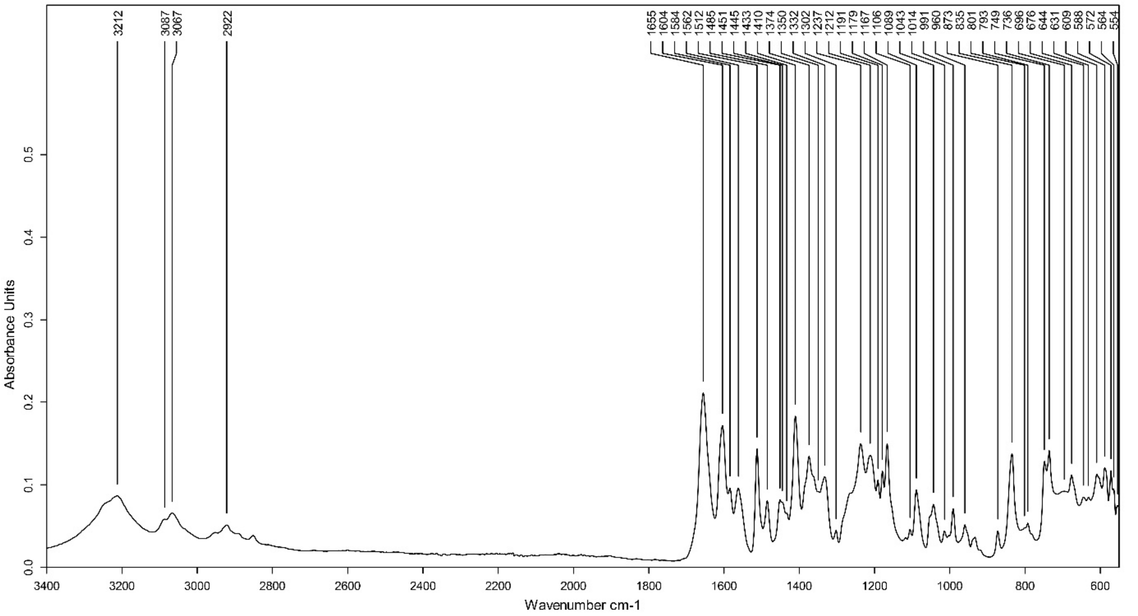

File : HN_pOH.0	Frequency Range : 499.546 - 3998.29	Measured on : 11/19/2018

Technique : ATR	Resolution : 2
Acquisition : Double Sided,Forward-Backw	Zerofilling : 2

Instrument : Tensor 27	Sample Scans : 64
Scan Time (sec) : 98.9919	Aperture : 6 mm

File path : D:\hn-IR

Sample : HN_pOH

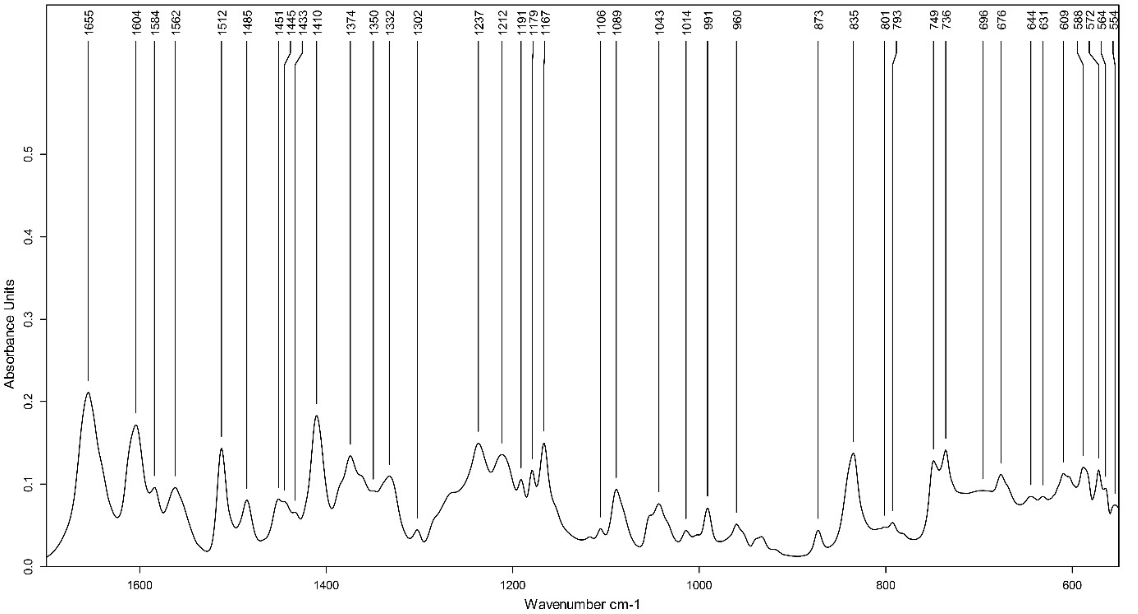

File : HN_pOH.0	Frequency Range : 499.546 - 3998.29	Measured on : 11/19/2018

Technique : ATR	Resolution : 2
Acquisition : Double Sided,Forward-Backw	Zerofilling : 2

Instrument : Tensor 27	Sample Scans : 64
Scan Time (sec) : 98.9919	Aperture : 6 mm

File path : D:\hn-IR

Figure S37. ATR-IR spectrum of **3g**

Sample : HN-2,3-diOH

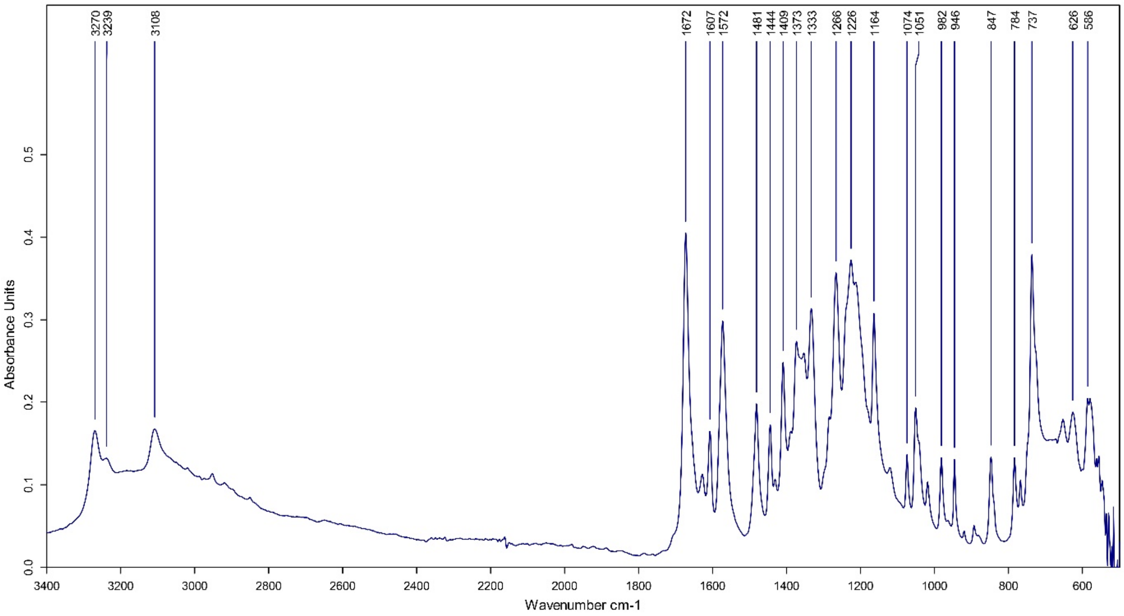

File : HN-2,3-diOH.0	Frequency Range : 499.546 - 3998.29	Measured on : 2/13/2019

Technique : ATR, hydrazone	Resolution : 2	Instrument : Tensor 27	Sample Scans : 32
Acquisition : Double Sided,Forward-Backw	Zerofilling : 2	Scan Time (sec) : 49.451	Aperture : 6 mm

File path : D:\IR\Neda

Sample : HN-2,3-diOH

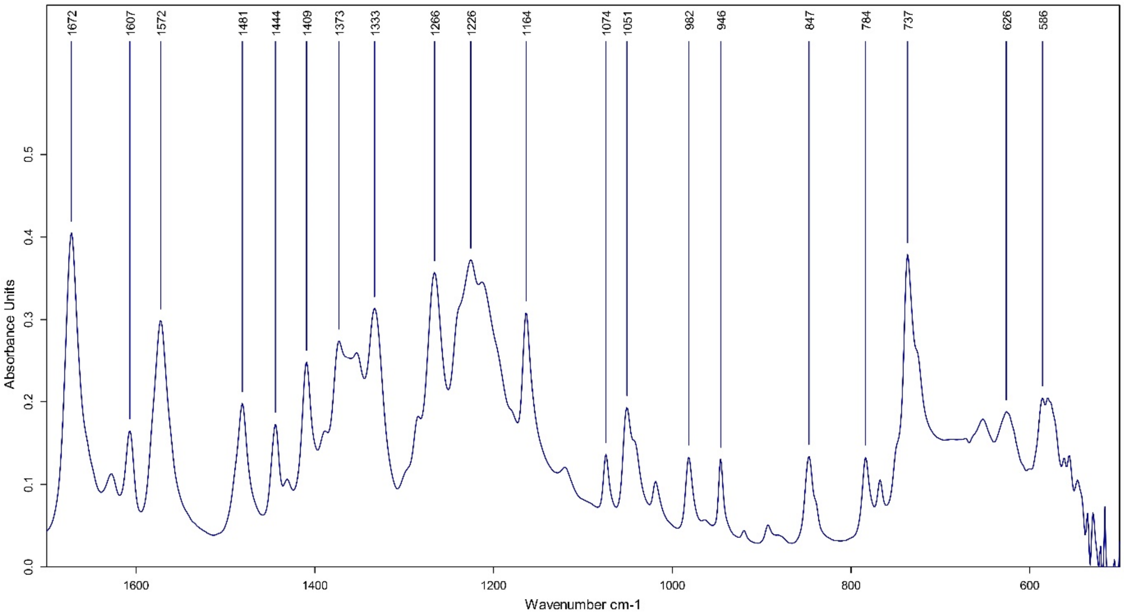

File : HN-2,3-diOH.0	Frequency Range : 499.546 - 3998.29	Measured on : 2/13/2019

Technique : ATR, hydrazone	Resolution : 2	Instrument : Tensor 27	Sample Scans : 32
Acquisition : Double Sided,Forward-Backw	Zerofilling : 2	Scan Time (sec) : 49.451	Aperture : 6 mm

File path : D:\IR\Neda

Figure S38. ATR-IR spectrum of **3h**

Sample : HN_diOH2_4

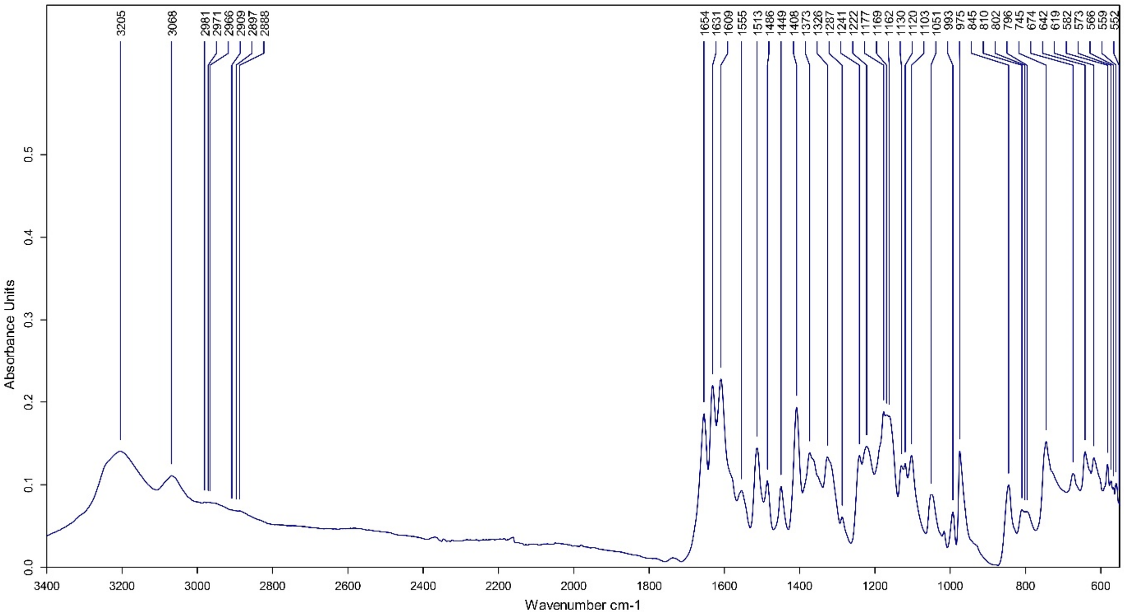

File : HN_diOH2_4.0	Frequency Range : 499.546 - 3998.29	Measured on : 12/13/2018

Technique : ATR	Resolution : 2	Instrument : Tensor 27	Sample Scans : 64
Acquisition : Double Sided,Forward-Backw	Zerofilling : 2	Scan Time (sec) : 98.996	Aperture : 6 mm

File path : D:\hn-IR

Sample : HN_diOH2_4

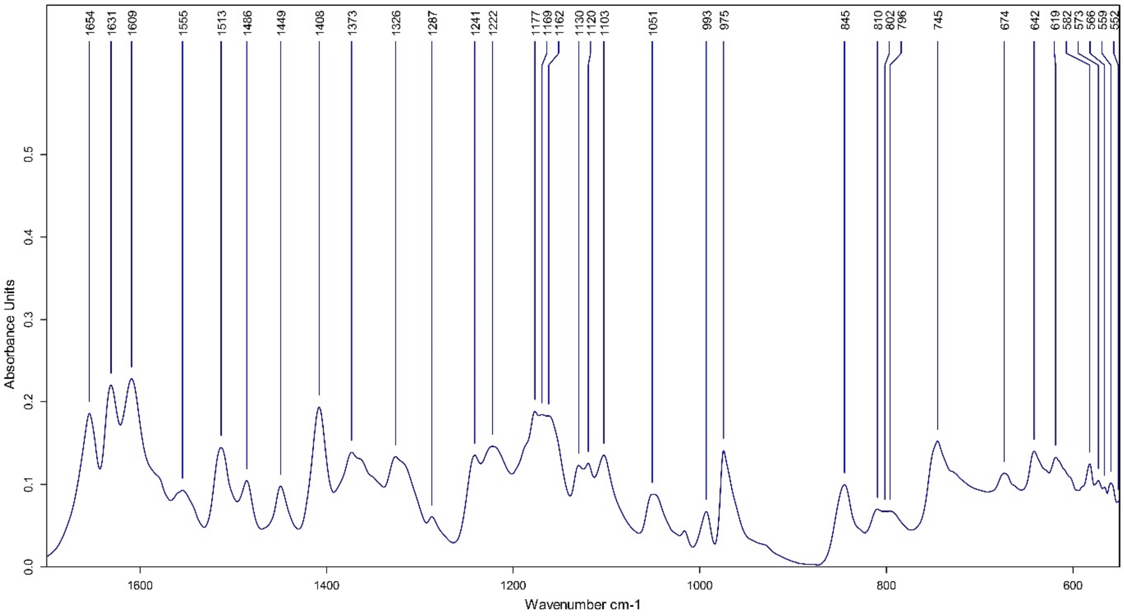

File : HN_diOH2_4.0	Frequency Range : 499.546 - 3998.29	Measured on : 12/13/2018

Technique : ATR	Resolution : 2	Instrument : Tensor 27	Sample Scans : 64
Acquisition : Double Sided,Forward-Backw	Zerofilling : 2	Scan Time (sec) : 98.996	Aperture : 6 mm

File path : D:\hn-IR

Figure S39. ATR-IR spectrum of **3i**

Sample : HN-3,4-diOH

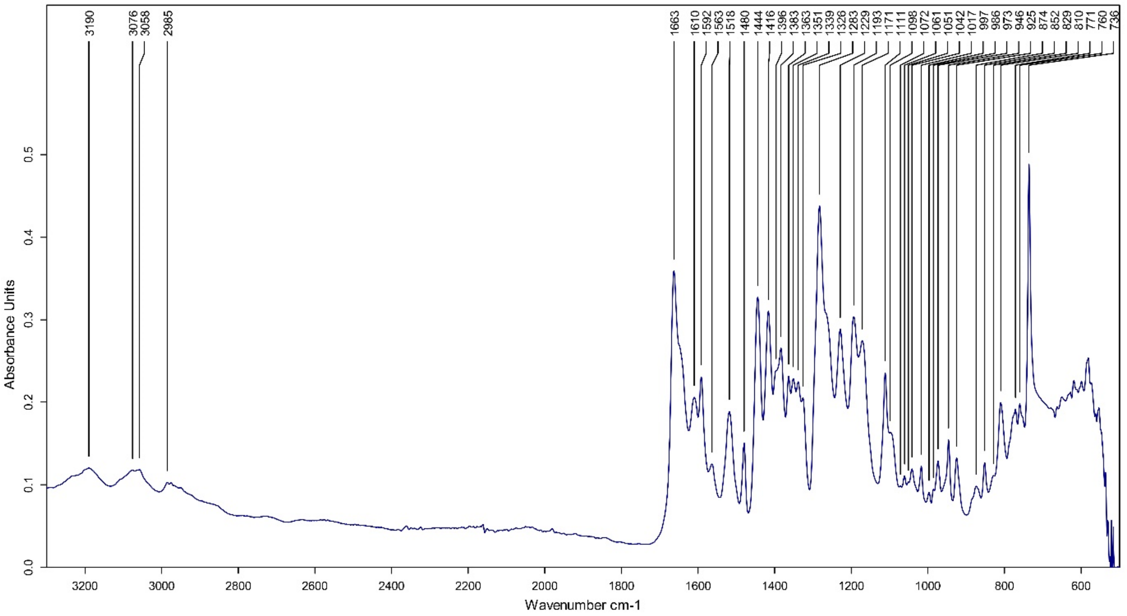

File : HN-3,4-diOH.0	Frequency Range : 499.546 - 3998.29	Measured on : 2/13/2019

Technique : ATR, hydrazone	Resolution : 2
Acquisition : Double Sided,Forward-Backw	Zerofilling : 2

Instrument : Tensor 27	Sample Scans : 32
Scan Time (sec) : 49.452	Aperture : 6 mm

File path : D:\IR\Neda

Sample : HN-3,4-diOH

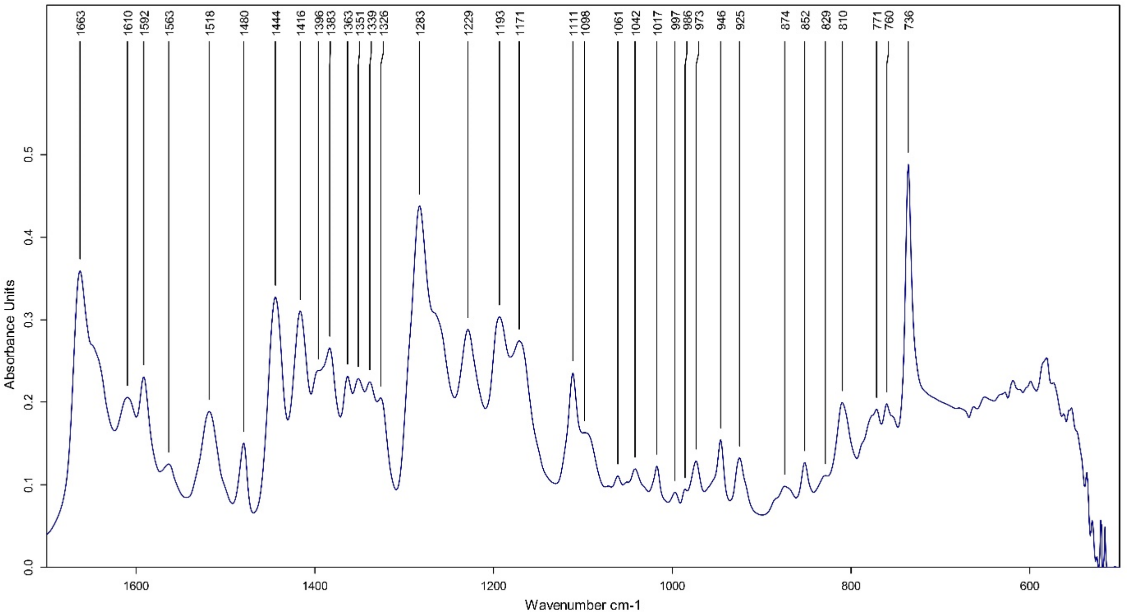

File : HN-3,4-diOH.0	Frequency Range : 499.546 - 3998.29	Measured on : 2/13/2019

Technique : ATR, hydrazone	Resolution : 2
Acquisition : Double Sided,Forward-Backw	Zerofilling : 2

Instrument : Tensor 27	Sample Scans : 32
Scan Time (sec) : 49.452	Aperture : 6 mm

File path : D:\IR\Neda

Figure S40. ATR-IR spectrum of **3j**

Sample : HN_234triOH

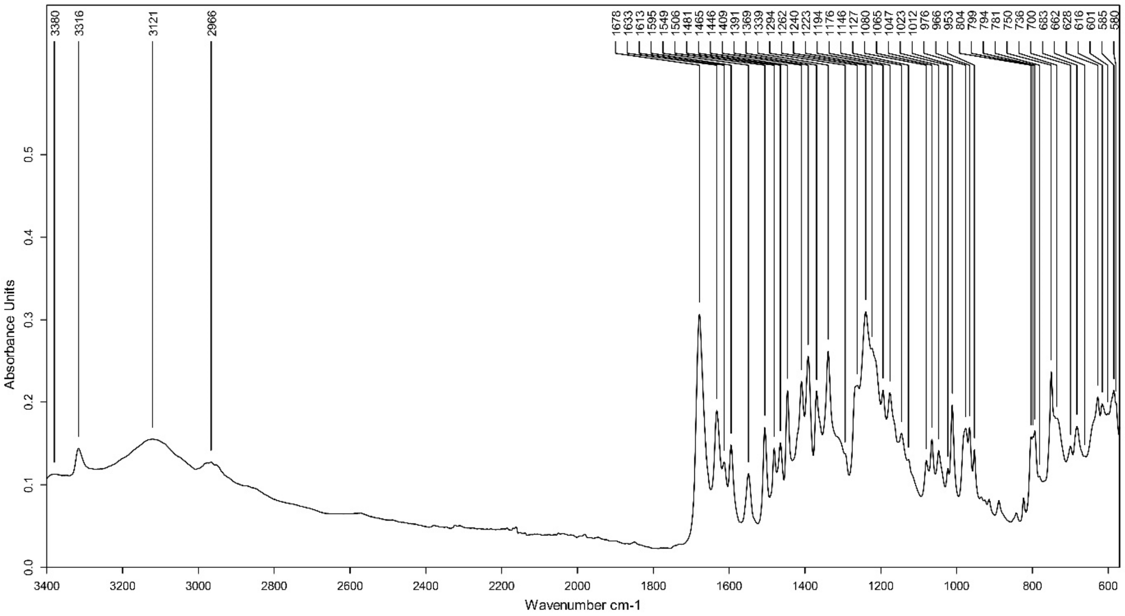

File : HN_234triOH.0	Frequency Range : 499.546 - 3998.29	Measured on : 4/15/2019

Technique : ATR, dizamesteno,	Resolution : 2	Instrument : Tensor 27	Sample Scans : 64
Acquisition : Double Sided,Forward-Backw	Zerofilling : 2	Scan Time (sec) : 98.9529	Aperture : 6 mm

File path : D:\hn-IR

Sample : HN_234triOH

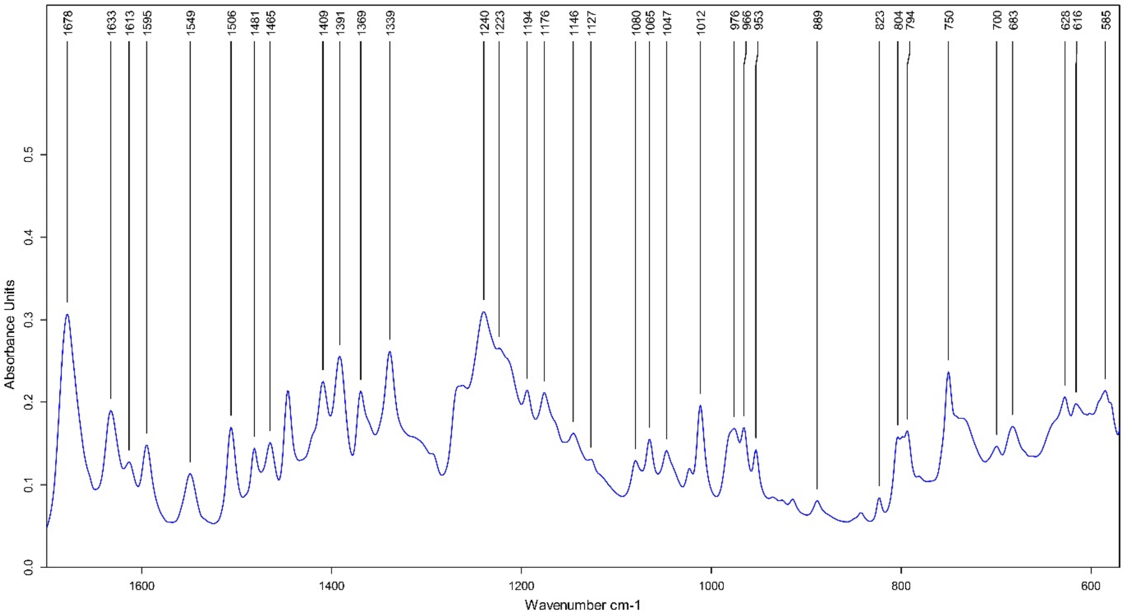

File : HN_234triOH.0	Frequency Range : 499.546 - 3998.29	Measured on : 4/15/2019

Technique : ATR, dizamesteno,	Resolution : 2	Instrument : Tensor 27	Sample Scans : 64
Acquisition : Double Sided,Forward-Backw	Zerofilling : 2	Scan Time (sec) : 98.9529	Aperture : 6 mm

File path : D:\hn-IR

Figure S41. ATR-IR spectrum of **3k**

Sample : HN-2-OH-3-OCH3

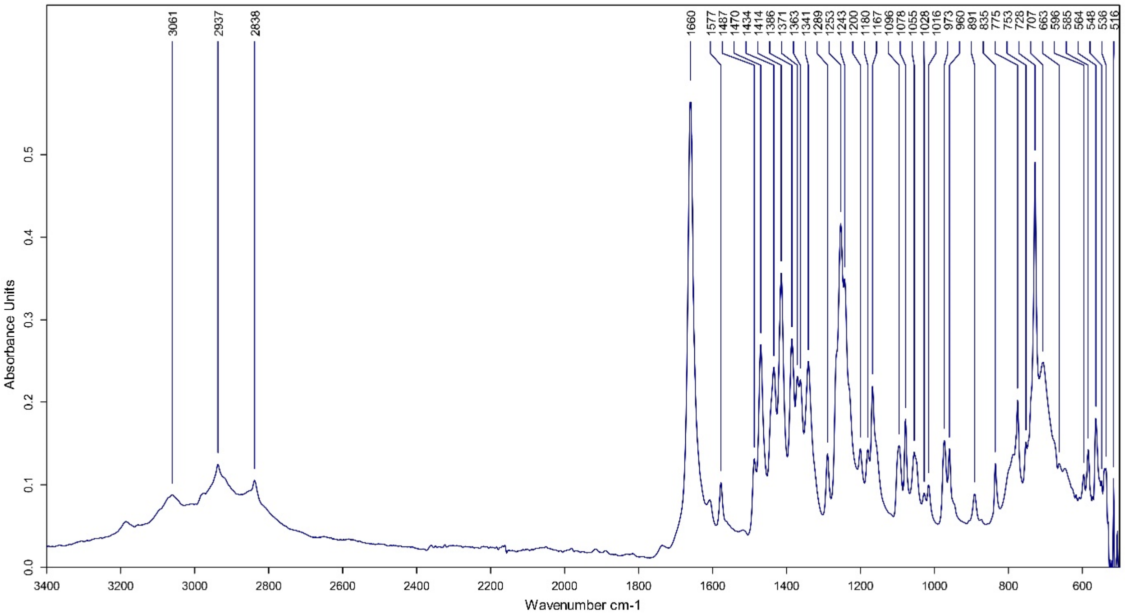

File : HN-2-OH-3-OCH3.0	Frequency Range : 499.546 - 3998.29	Measured on : 2/13/2019

Technique : ATR, hydrazone	Resolution : 2	Instrument : Tensor 27	Sample Scans : 32
Acquisition : Double Sided,Forward-Backw	Zerofilling : 2	Scan Time (sec) : 49.452	Aperture : 6 mm

File path : D:\IR\Neda

Sample : HN-2-OH-3-OCH3

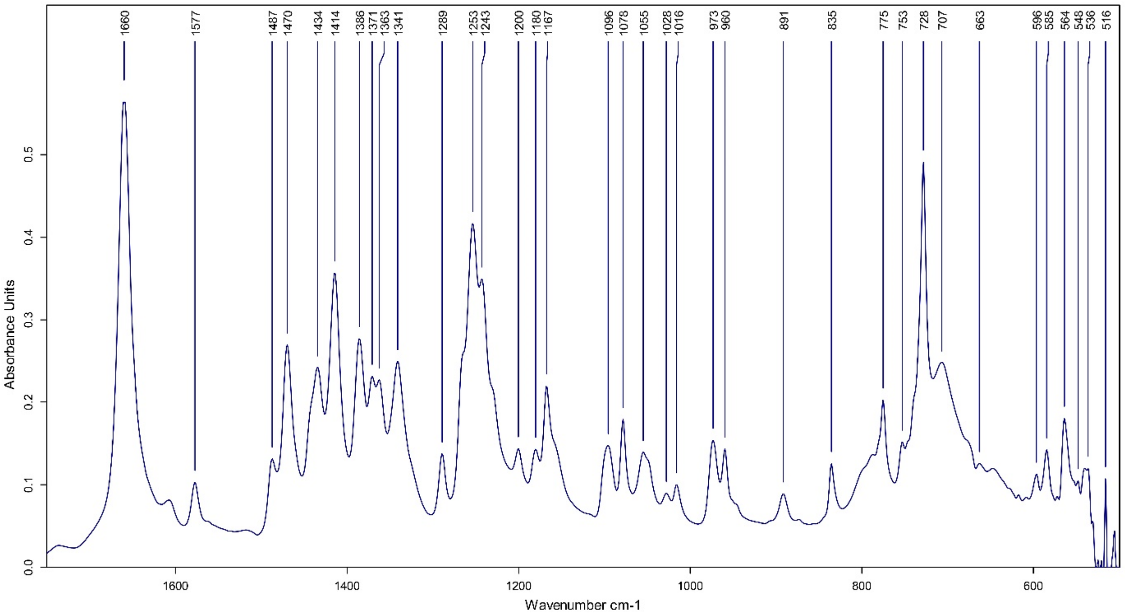

File : HN-2-OH-3-OCH3.0	Frequency Range : 499.546 - 3998.29	Measured on : 2/13/2019

Technique : ATR, hydrazone	Resolution : 2	Instrument : Tensor 27	Sample Scans : 32
Acquisition : Double Sided,Forward-Backw	Zerofilling : 2	Scan Time (sec) : 49.452	Aperture : 6 mm

File path : D:\IR\Neda

Figure S42. ATR-IR spectrum of **3l**

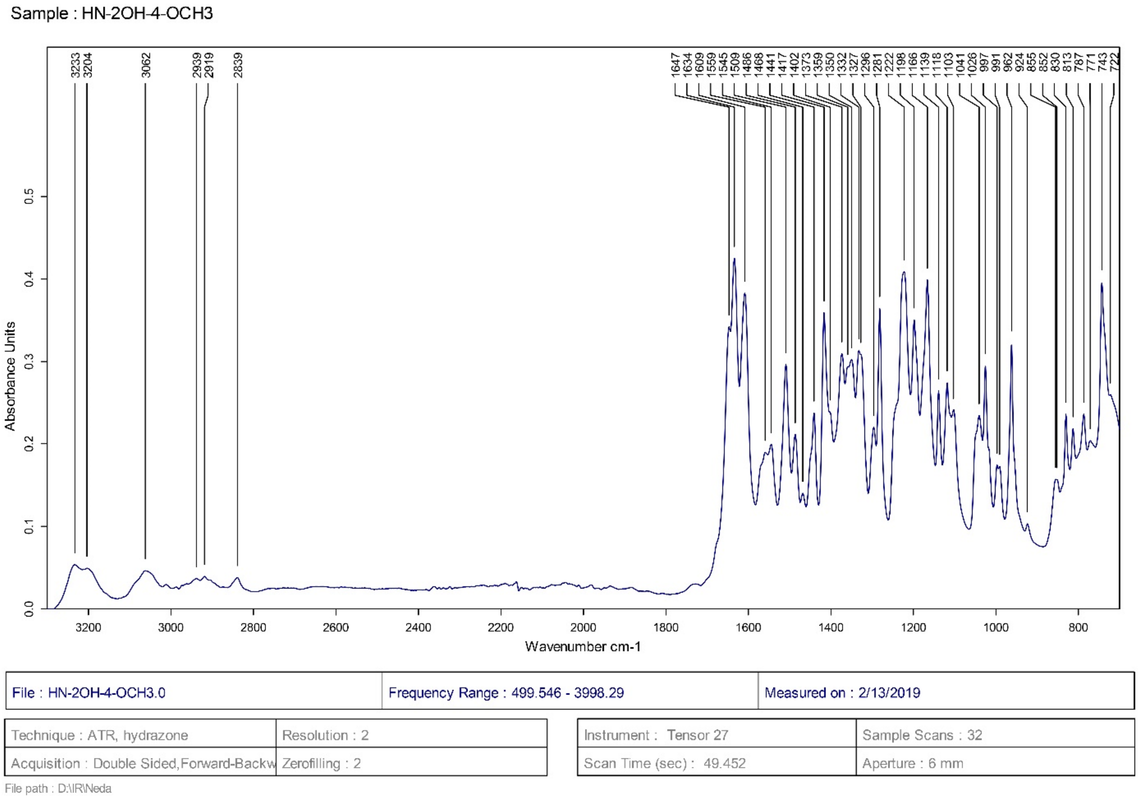

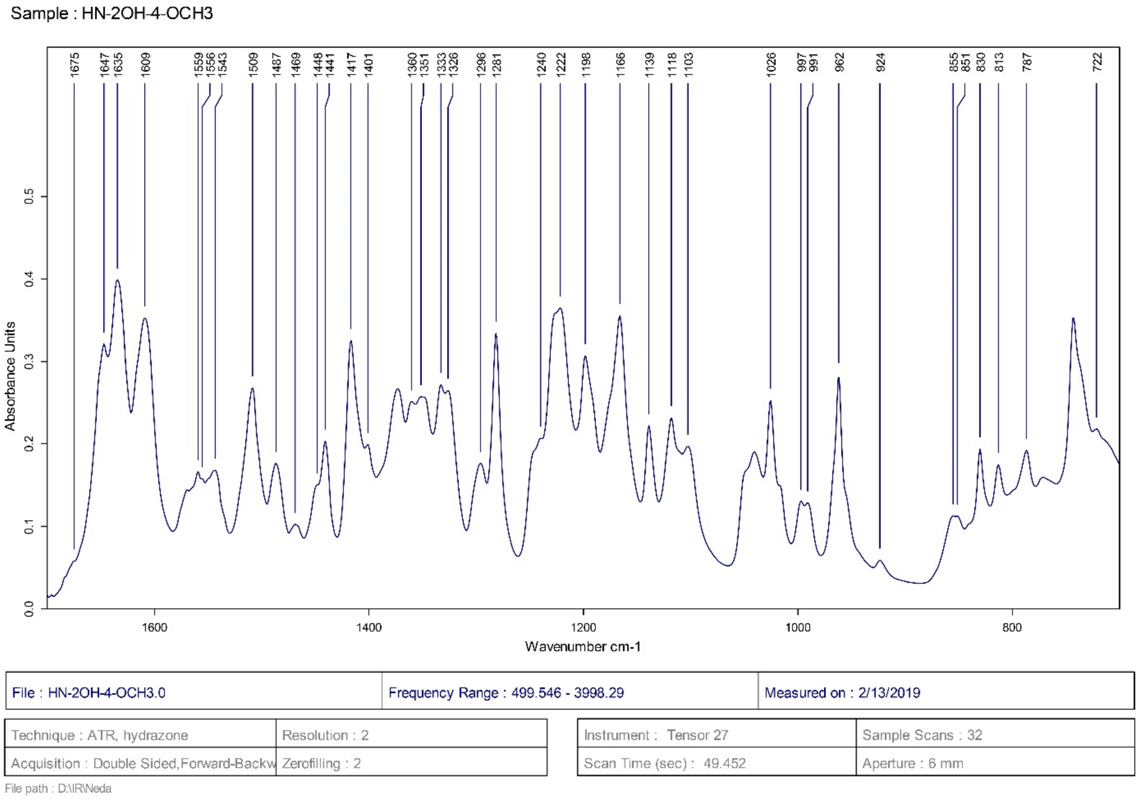

Figure S43. ATR-IR spectrum of **3m**

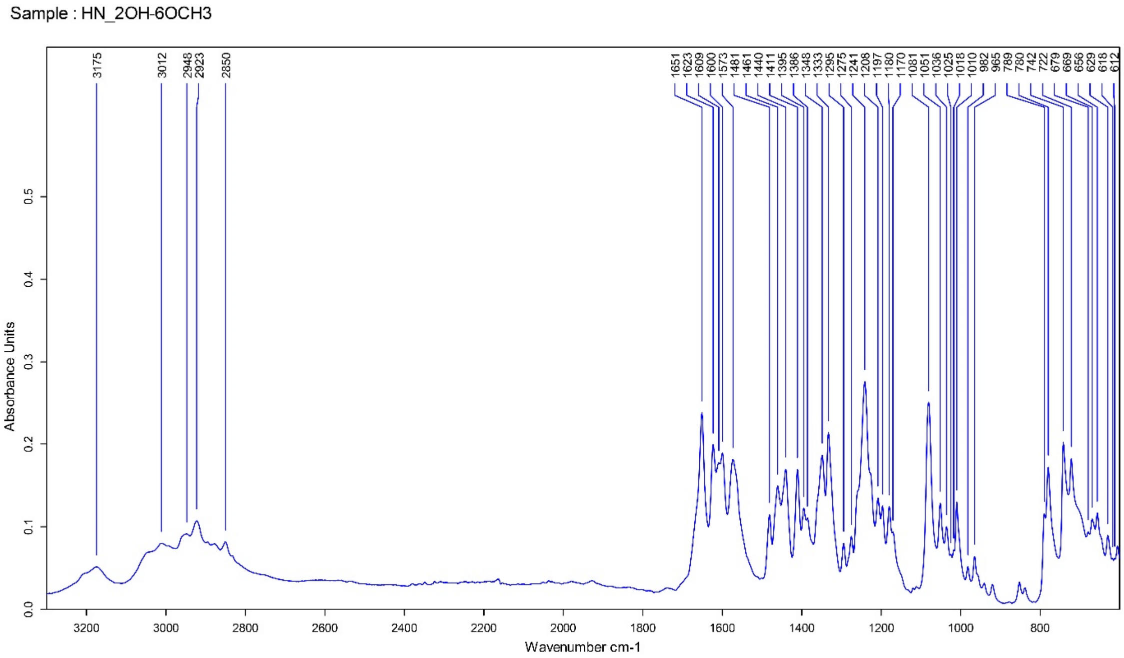

File : HN_2OH-6OCH3.0	Frequency Range : 499.546 - 3998.29	Measured on : 3/22/2019

Technique : ATR, hartiq, tush	Resolution : 2	Instrument : Tensor 27	Sample Scans : 64
Acquisition : Double Sided,Forward-Backw	Zerofilling : 2	Scan Time (sec) : 98.95	Aperture : 6 mm

File path : D:\hn-IR

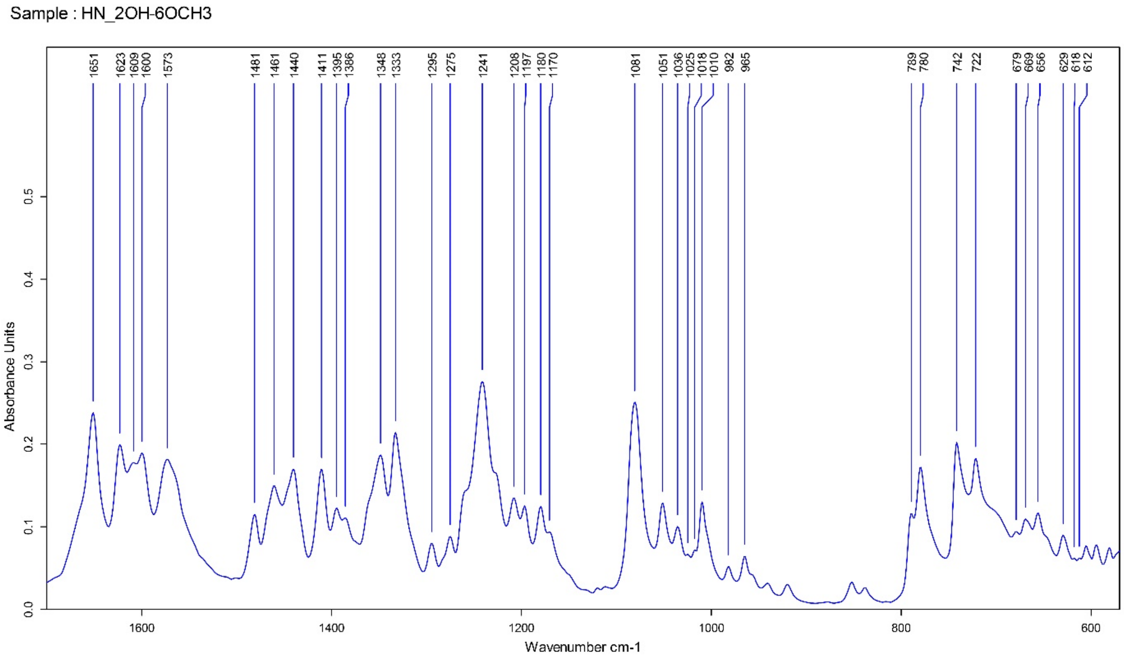

File : HN_2OH-6OCH3.0	Frequency Range : 499.546 - 3998.29	Measured on : 3/22/2019

Technique : ATR, hartiq, tush	Resolution : 2	Instrument : Tensor 27	Sample Scans : 64
Acquisition : Double Sided,Forward-Backw	Zerofilling : 2	Scan Time (sec) : 98.95	Aperture : 6 mm

File path : D:\hn-IR

Figure S44. ATR-IR spectrum of **3n**

Sample : HN-3-OH-4-OCH3

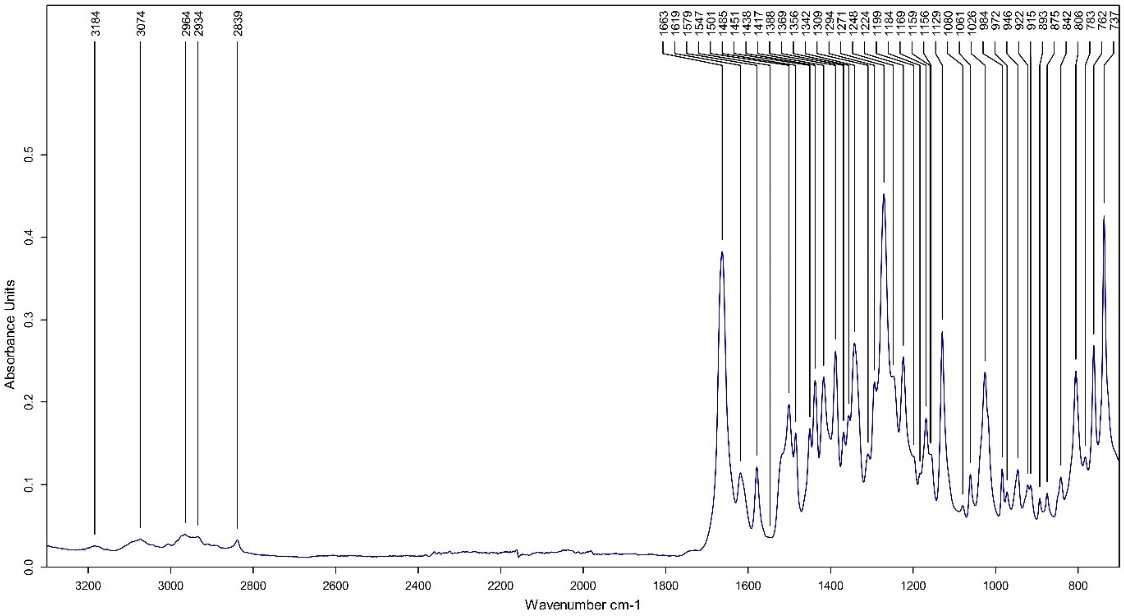

File : HN-3-OH-4-OCH3.0	Frequency Range : 499.546 - 3998.29	Measured on : 2/13/2019

Technique : ATR, hydrazone, NH-diOH, dv	Resolution : 2	Instrument : Tensor 27	Sample Scans : 32
Acquisition : Double Sided,Forward-Backw	Zerofilling : 2	Scan Time (sec) : 49.452	Aperture : 6 mm

File path : D:\IR\Neda

Sample : HN-3-OH-4-OCH3

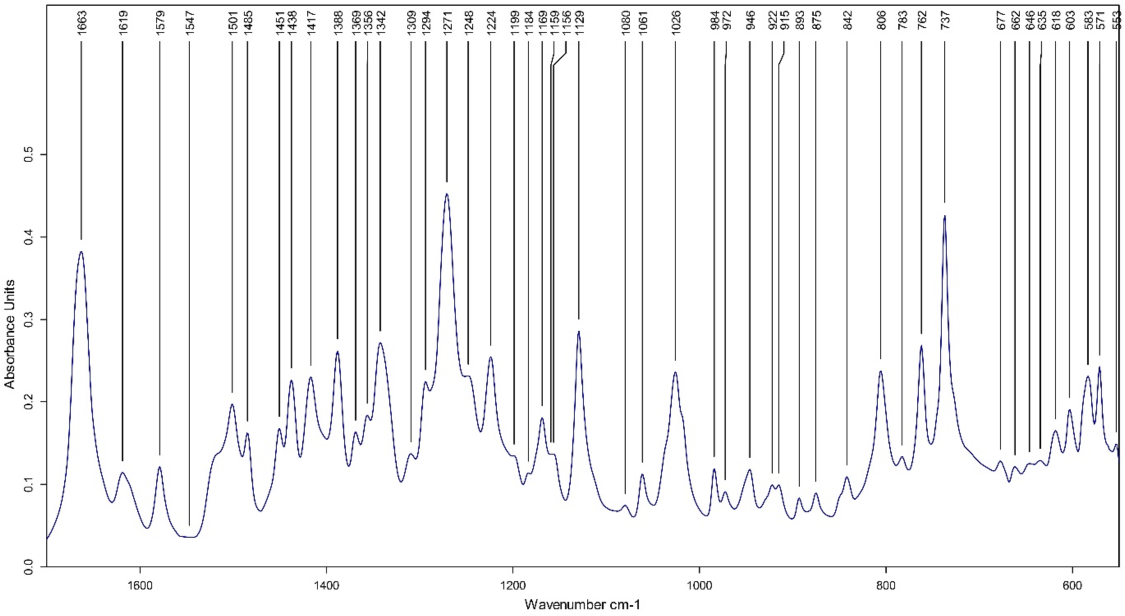

File : HN-3-OH-4-OCH3.0	Frequency Range : 499.546 - 3998.29	Measured on : 2/13/2019

Technique : ATR, hydrazone, NH-diOH, dv	Resolution : 2	Instrument : Tensor 27	Sample Scans : 32
Acquisition : Double Sided,Forward-Backw	Zerofilling : 2	Scan Time (sec) : 49.452	Aperture : 6 mm

File path : D:\IR\Neda

Figure S45. ATR-IR spectrum of **3o**

Sample : HN_4OH_2OCH3

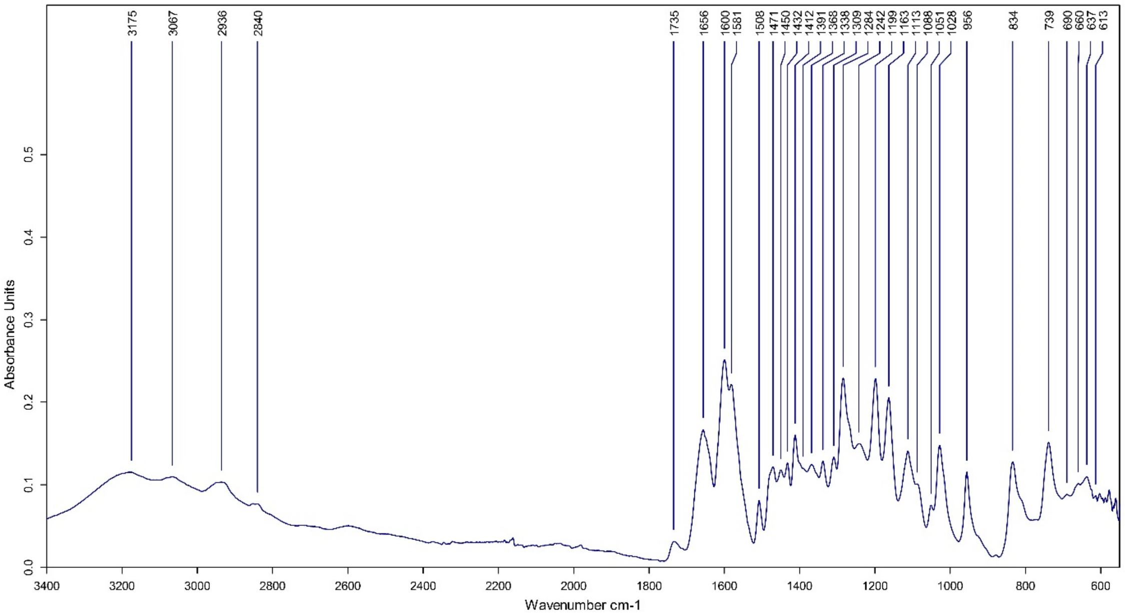

File : HN_4OH_2OCH3.0	Frequency Range : 499.546 - 3998.29	Measured on : 5/27/2020

Technique : ATR, HN_4OH_2OCH3_27.05	Resolution : 2	Instrument : Tensor 27	Sample Scans : 64
Acquisition : Double Sided,Forward-Backw	Zerofilling : 2	Scan Time (sec) : 98.931	Aperture : 6 mm

File path : D:\hn-IR

Sample : HN_4OH_2OCH3

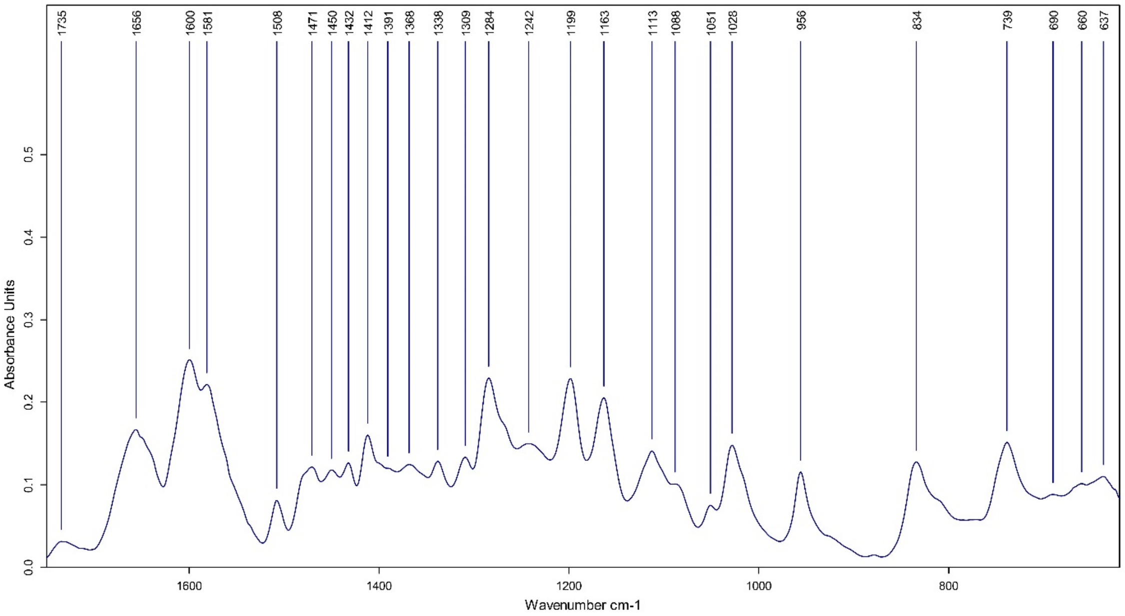

File : HN_4OH_2OCH3.0	Frequency Range : 499.546 - 3998.29	Measured on : 5/27/2020

Technique : ATR, HN_4OH_2OCH3_27.05	Resolution : 2	Instrument : Tensor 27	Sample Scans : 64
Acquisition : Double Sided,Forward-Backw	Zerofilling : 2	Scan Time (sec) : 98.931	Aperture : 6 mm

File path : D:\hn-IR

Figure S46. ATR-IR spectrum of **3p**

Sample : HN_diOH_5OCH3

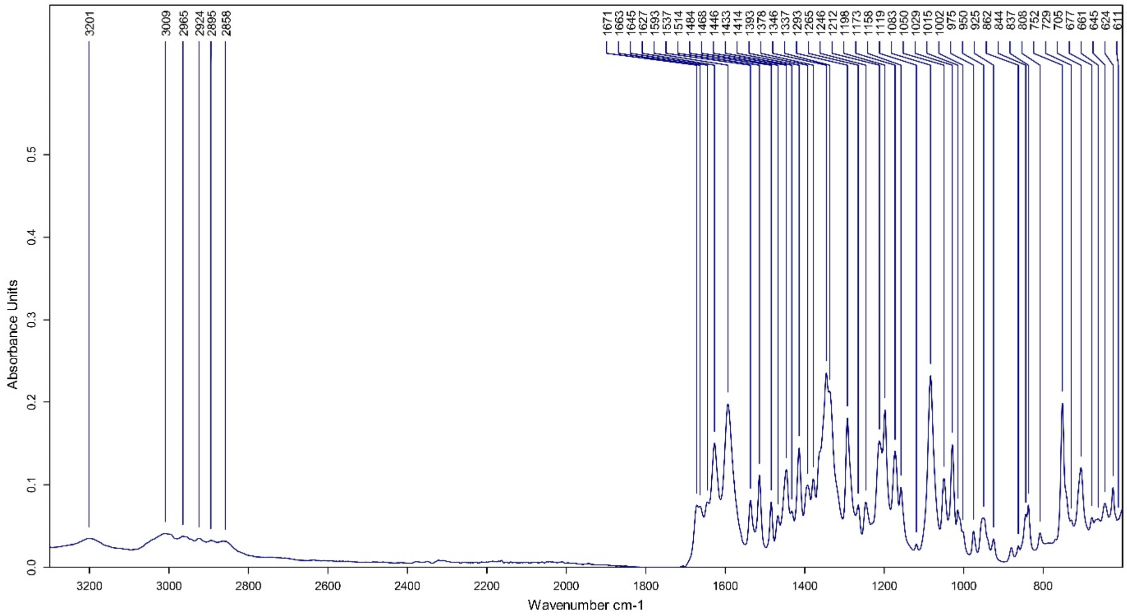

File : HN_diOH_5OCH3.0	Frequency Range : 499.546 - 3998.29	Measured on : 4/15/2019

Technique : ATR, dizamesteno, 3,4diOH-5	Resolution : 2	Instrument : Tensor 27	Sample Scans : 64
Acquisition : Double Sided,Forward-Backw	Zerofilling : 2	Scan Time (sec) : 98.952	Aperture : 6 mm

File path : D:\hn-IR

Sample : HN_diOH_5OCH3

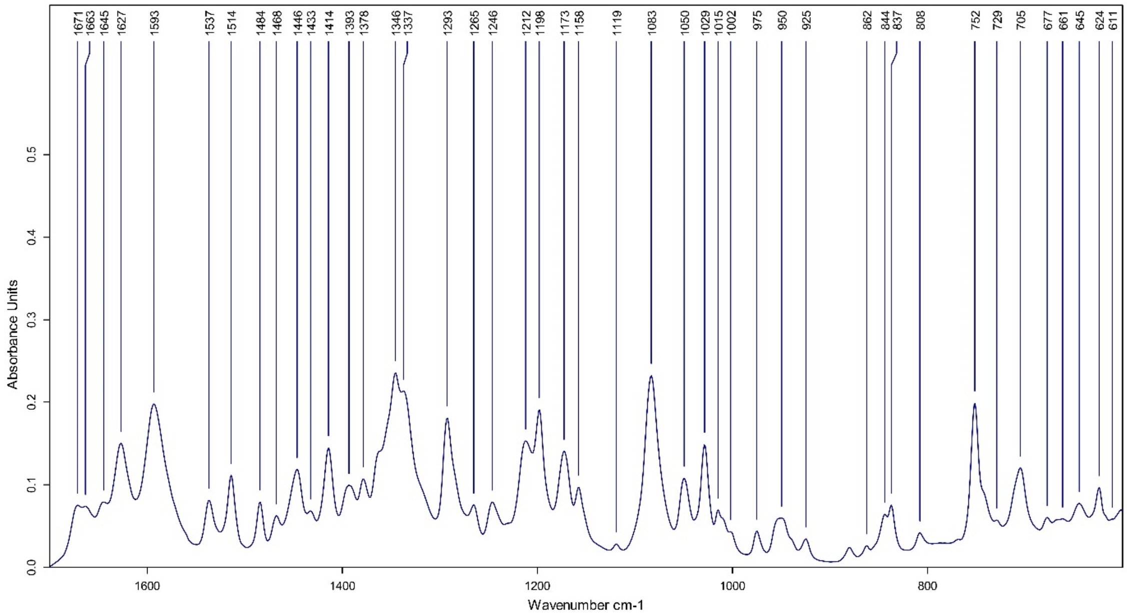

File : HN_diOH_5OCH3.0	Frequency Range : 499.546 - 3998.29	Measured on : 4/15/2019

Technique : ATR, dizamesteno, 3,4diOH-5	Resolution : 2	Instrument : Tensor 27	Sample Scans : 64
Acquisition : Double Sided,Forward-Backw	Zerofilling : 2	Scan Time (sec) : 98.952	Aperture : 6 mm

File path : D:\hn-IR

Figure S47. ATR-IR spectrum of **3q**

4. Three-dimensional (3D) representation of lower-energy docking poses of 3h in the MAO-B cavity

(A) Binding pose of **3h** in a conformation featuring hydrogen bonds between the hydroxyl groups and the o-OH groups and the N-azomethyne atoms

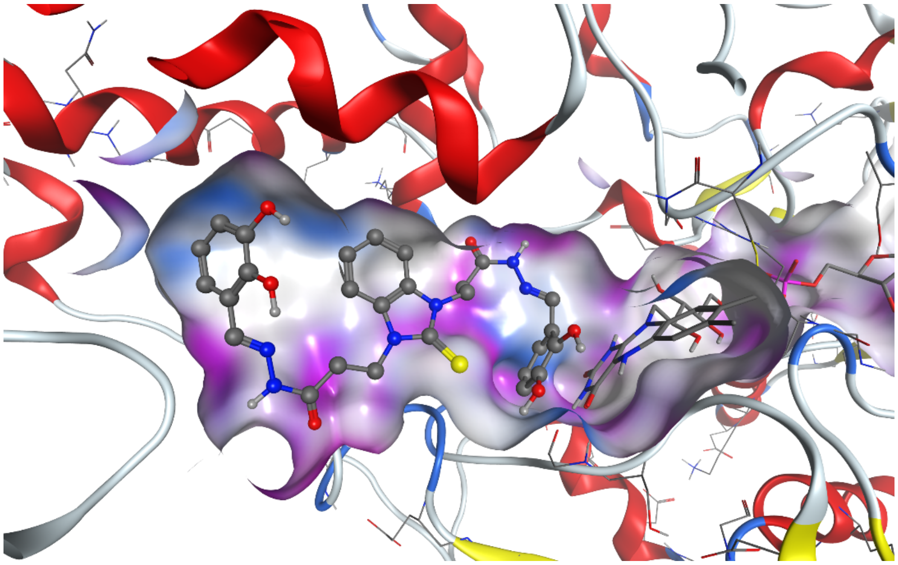

Interaction energy: 1.073 kcal.mol^{-1} lower than the best binding pose

(B) Binding pose of **3h** in a conformation featuring hydrogen bonds between the hydroxyl groups

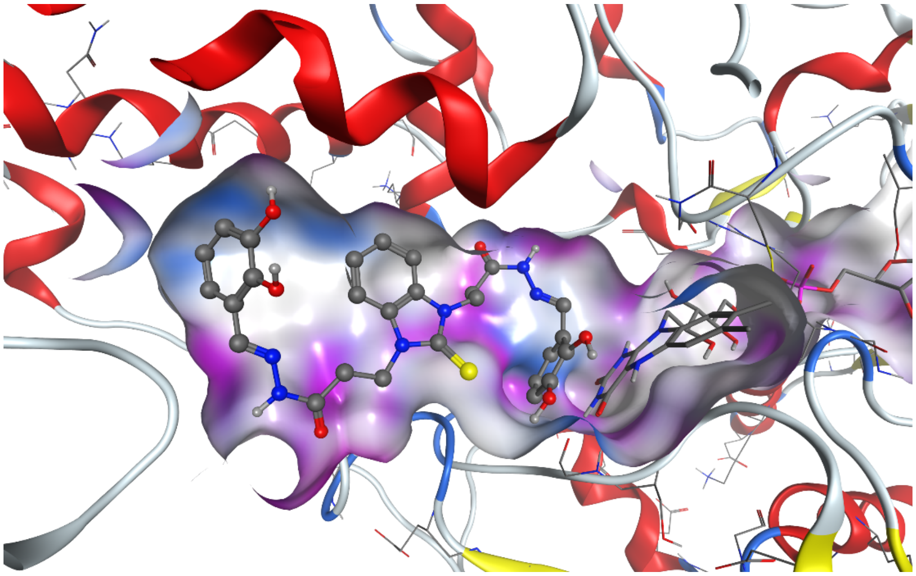

Interaction energy: 1.100 kcal.mol^{-1} lower than the best binding pose

(C) Binding pose of **3h** in a conformation featuring hydrogen bonds between the o-OH groups and the azomethyne N-atoms

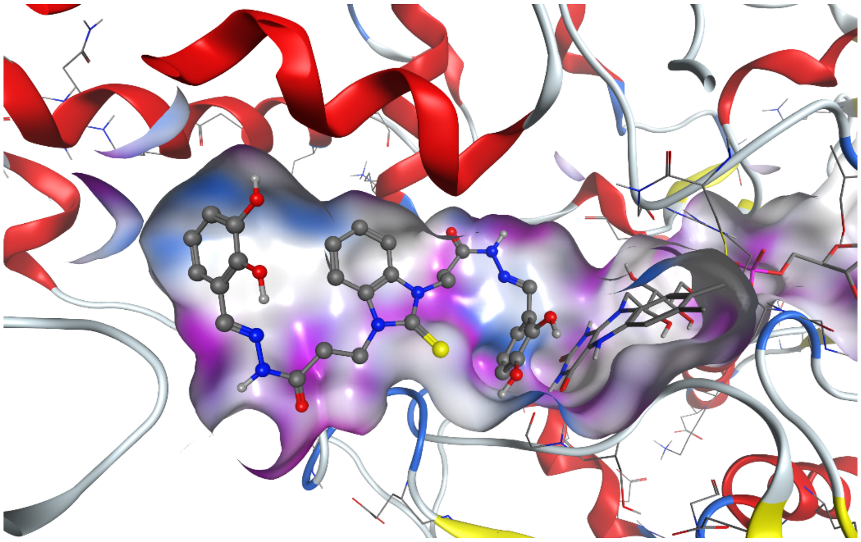

Interaction energy: 1.118 kcal.mol^{-1} lower than the best binding pose

Figure S48. Three-dimensional (3D) representation of the interactions of lower-energy docking poses of 3h in the MAO-B cavity. Docked structures are represented by the balls and sticks method, while FAD is represented by sticks.
